# MMBGR Protocol - infants and preschoolers: Instructive and Orofacial Myofunctional Clinical History

**DOI:** 10.1590/2317-1782/20212020324

**Published:** 2022-01-10

**Authors:** Andréa Monteiro Correia Medeiros, Irene Queiroz Marchesan, Katia Flores Genaro, Íkaro Daniel de Carvalho Barreto, Giédre Berretin-Felix

**Affiliations:** 1 Departamento de Fonoaudiologia, Universidade Federal de Sergipe – UFS - São Cristóvão (SE), Brasil.; 2 CEFAC Saúde e Educação - São Paulo (SP), Brasil.; 3 Departamento de Fonoaudiologia, Faculdade de Odontologia de Bauru, Universidade de São Paulo – FOB/USP - Bauru (SP), Brasil.; 4 Departamento de Estatística e Informática, Universidade Federal Rural de Pernambuco – UFRPE - Recife (PE), Brasil

**Keywords:** Speech, Language and Hearing Sciences, Infant, Preschool, Validation Studies, Medical History Taking, Diagnosis, Myofunctional Therapy, Stomatognathic System

## Abstract

**Purpose:**

To present Myofunctional Orofacial Clinical History Instructive and Protocol belonging to the MMBGR Protocol - Infants and Preschoolers, including the adaptation and validation of content and appearance.

**Methods:**

Validation, descriptive and cross-sectional study. Adaptation based on the MBGR Protocol, based on theoretical studies and the authors' experience. Infants between 6 and 23 months of age and preschoolers between 24 and 71 months were considered. Consent and consensus of the version adapted by the authors (original and current) was obtained. The appearance and content of the new instrument were analyzed by 10 speech therapists specialized in Orofacial Motricity. We performed two analysis rounds. First: an electronic form containing dichotic questions (yes / no), with justification for negative answers; Content Validity Index and Exact Binomial Test; Second: 5 points-Likert scale.

**Results:**

We produced an unprecedented instructional and adapted Clinical History protocol maintaining 23 age group related items. We excluded information from 7 items and included information in 8. Initially, we achieved agreement in 70% instructional specialist items by at least 70% of the specialists; and 64% of Clinical History items, by at least 90% of specialists. In the second round, there were 100% of “I totally agree” responses from the experts.

**Conclusion:**

“Instructive” and “Myofunctional Clinical History, Orofacial” had validity of content and appearance concluded, and together with the “Clinical Examination” they integrate the “Protocol MMBGR - Infants and Preschoolers”, being able to contribute to clinical practice and research in Motricity Orofacial area in the age group between 6 months and 5 years and 11 months of age.

## INTRODUCTION

Understanding the development of orofacial myofunctional aspects at an early age can contribute to a better understanding of the normal functioning of the stomatognathic system and early intervention aimed at any changes. Infants are defined as being between 1 and 24 months old, and preschoolers are defined as being between 2 and 5 years old, according to Health Area Descriptors (DECs).

The clinical history survey in Speech-Language Pathology is critical for recognizing and understanding the problem that involves the individual and his/her family, while also allowing elucidation of specific questions about the development of Orofacial Motricity (OM), which are necessary to guide the situation of the clinical examination and establish clinical reasoning for the therapeutic intervention.

The rescue of orofacial function development in young children, using standardized and validated instruments, is part of the parents' report via a family survey^(1–3)^. The approximation between the speech therapist and the family can be done objectively or subjectively ^(4)^, depending on the theoretical assumptions that support therapeutic understanding and guide the speech therapist's clinical practice.

The use of protocols has been appointed as an important tool for recording and monitoring clinical care, but they are still scarce in speech therapy^(5)^, where more research is needed to propose the organization of validated materials with reliable psychometric properties^(6)^ for the OM clinic.

The following steps are considered in the validation process of tests in Speech-Language Pathology and Audiology: Validity Evidence based on content, internal consistency and relationship with other variables; Validity Evidence based on response processes; Reliability/Accuracy; Equity; Accuracy; and Validity Evidence based on test consequences^(7)^.

The main instruments that have been used in the OM area are the Orofacial Myofunctional Assessment with Scores – OMES protocols^(6,8)^ and the MBGR Protocol ^(9,10)^, both aimed at the population over 6 years of age. There are also protocols for the neonatal age group, which address breastfeeding, breastfeeding readiness and OM ^(11–14)^. For infants, the SOMA (Schedule for Oral Motor Assessment) is highlighted^(15)^ on oral motor skills should be highlighted; and for infants and preschoolers, the PAD-PET ^(16)^, which addresses risk for dysphagia.

Given the scarcity of national instruments for the age groups of Brazilian infants and preschoolers, the need arose to adapt and validate the MBGR Protocol ^(10)^ for use in the age group from 6 months to 5 years and 11 months of life, having been chosen for its scope and specificity in the OM area, taking into account the research purpose of both the Clinical History and the Orofacial Myofunctional Clinical Examination, in addition to the construction of the respective instruction.

The goal of this article is to present the "Instructive" and "Orofacial Myofunctional Clinical History" Protocols that comprise the "MMBGR Protocol - Infants and Preschoolers," demonstrating the adaptation process and respective validation based on test content analysis.

## METHODS

It is a validation, descriptive, and cross-sectional study that was conducted between May and December 2019 as part of a larger project that was approved by the Ethics and Research in Human Beings Committee of the Universidade Federal de Sergipe under protocol number CAAE 12529419.6.0000.5546. The Informed Consent Form was signed by all those responsible for the research participants.

This is the validation of a new instrument, adapted from the MBGR ^(10)^, intended for the infant and preschool population, in accordance with the guidelines recommended in validation studies, taking into account the stage based on the test content ^(7)^, after a written opinion favorable to the adaptation of the protocol by the authors of the original MBGR protocol.

Initially, a theoretical study on orofacial myofunctional development and stomatognathic functions at early ages was conducted using a search on the Scielo, Pubmed, and Bireme platforms from 1993 to 2017. The descriptors "Speech Therapy", "Infants", "Pre-Schools", "Assessment Methods", and "Stomatognathic System" taking into account full-text periodicals and dissertations/theses, with an emphasis on protocols in the OM area validated in Brazil. In terms of knowledge of existing instruments, we relied on the authors' expertise.

The instructional guide was developed, and an adapted version of the “Orofacial Myofunctional Clinical History” was prepared, taking syntactic and semantic aspects into account. These were submitted for consideration to the original authors, and after the suggestions were revised and consensus was reached among the authors (original and current versions), the appearance and content of the new instrument were analyzed.

This stage involved 10 OM-specialized speech therapists from four regions of Brazil (Center-West, Northeast, Southeast, and South), who were invited to participate in the study via e-mail and/or messaging application. An explanation letter was sent out, outlining the purpose of the work, the analysis, and the estimated time to complete the electronic form, along with the access link, which contained specific instructions on how to evaluate each item and the instrument as a whole.

The majority of specialists (70%) were between the ages of 41 and 50 years old; all had a postgraduate degree (80% Doctorate and 20% Master's) and experience in the OM field, most for more than 15 years (90%) and with teaching experience for at least five years; and all were chosen because they work with infants (80%) and preschoolers (80%), as shown in Table 1.

**Table 1:** Sociodemographic and professional characterization of specialist speech therapists

	**N**	**%**
**Age group**		
Between 31 and 40 years old	3	30,0
Between 41 and 50 years old	7	70,0
**Currently, in which region of the country do you practice the profession:**		
Center-West	2	20,0
Northeast	3	30,0
Southeast	1	10,0
South	4	40,0
**What is your degree?**		
Doctor	8	80,0
Master	2	20,0
**Teaching. For how long?**		
Less than 5 years	1	10,0
Between 5 and 10 years old	3	30,0
Between 10 and 15 years old	1	10,0
Between 15 and 20 years old	3	30,0
Between 25 and 30 years old	2	20,0
**Years of experience in Speech Therapy – Orofacial Motricity.**		
Between 5 and 10 years old	1	10,0
Between 15 and 20 years old	4	40,0
Between 20 and 25 years old	5	50,0
**Do you work in the area of Orofacial Motricity with Infants (up to 24 months of life)?**	8	80,0
**For how long?**		
Less than 5 years	1	12,5
Between 5 and 10 years old	2	25,0
Between 10 and 15 years old	3	37,5
Between 15 and 20 years old	2	25,0
**Do you work in the area of Orofacial Motricity with preschoolers (24 months to 5 years of age)?**	8	80,0
**For how long?**		
Between 5 and 10 years old	2	25,0
Between 10 and 15 years old	1	12,5
Between 15 and 20 years old	1	12,5
Between 20 and 25 years old	4	50,0

**Caption:** n = absolute frequencies; % = percent

As an inclusion criterion, the specialist speech therapist should have at least five years of experience and/or teaching activity in speech therapy, as well as degrees and/or publications related to the OM area, and expertise in the subject, working with infants and/or preschoolers. Concerning the exclusion criteria, incomplete opinions and experts who did not deliver them by the deadlines (about 15 days for analysis, at each stage).

The new instrument was evaluated for the first time by speech therapists using an electronic form with dichotic questions (yes/no) and fields to justify negative answers. Thus, in the event of disagreement with a specific item, there were spaces available to describe which aspect was not in agreement and to suggest some changes.

The Content Validity Index (per item) and the exact binomial test were used for statistical analysis, with a minimum level of agreement of 70%. The Likert scale with five options was used in the second analysis of the instrument by the same experts (strongly agree, agree, indifferent, disagree and strongly disagree).

## RESULTS

The MBGR protocol was deemed appropriate for public use from 6 months to 5 years and 11 months of age, as its original version is intended for older age groups and has been indicated to be applied to children, adolescents, adults, and seniors^(10)^. The results presented here refer to the "Instructive" and "Orofacial Myofunctional Clinical History", which are part of the "MMBGR Protocol - Infants and Preschoolers".

The instructive was completely created and proposed to compose the new instrument known as the “MMBGR Protocol-Infants and Preschoolers,” in reference to the target age group, and with the addition of the letter M, an acronym for the researcher's surname and the main author of this version. In the adaptation of the MBGR "Orofacial Myofunctional Clinical History" protocol^(10)^ for infants and preschoolers, 23 items pertinent to the age group in question were maintained, information was excluded in 7 items and added in 8 items, which will be described below:

Maintained items related to Identification Data; Main complaint and other complaints; family history; Complications; Development and motor difficulties; Health problems; Breathing problems; Sleep; Treatments; Breastfeeding; Feed – introduction and current feed; Chewing; Swallowing; Habits – oral, biting and posture; Speech; Communication; Hearing; Voice; and Additional information.

Added items on Personal Data - Siblings (age of siblings); Motor Development (if you have already performed this motor skill, at what age, in addition to having included in the protocol information about the behavior that is expected by age group), which motor skills changed (holding head/rolling/sitting/crawling/staying standing/walking without support); Treatments (functional jaw orthopedics); Feeding development (feeding pattern and age group); Breastfeeding (beak); Feeding (difficulty in introducing utensils); Communication (sounds are absent).

Items not relevant to the age group covered, such as: Identification data on marital status, study, work, physical activity, were excluded; Complaints related to learning, jaw movements, and shoulder pain; frequency of motor difficulty; frequency of respiratory problems; aspects of dental treatment (implantation, extraction, prosthesis); oral habits (cigarette, pipe); and education. The modifications made to the Protocol "Orofacial Myofunctional Clinical History" are described in Table 2.

**Table 2:** Items in the Clinical History were changed to appear in the MMBGR based on the researcher's and the MBGR instrument's authors' considerations

**ITEM – MBGR –** **Clinical History (original)**	**MMBGR – Clinical History (modified items from MBGR)**
Personal Data	“Goes to daycare/school” / “class” instead of “works/has worked”/ “year”
How and where do you eat?	“Playing” instead of “Doing a lesson”
Chewing Capacity	Degree of satisfaction "of the family", rather than the degree of satisfaction of the "patient"
Communication	"Elaborate" instead of "It took time to elaborate"

In the first analysis of experts, 70% of the instructions obtained agreement from at least 70% of them; and 64% of the items in the “Clinical History” were in agreement from at least 90% (Table 3). For the second analysis, the final version of the instrument was submitted with all the suggested changes. Therefore, 100% of the responses “I totally agree” were obtained. The Instructive (Appendix 1) and the Protocol of “Orofacial Myofunctional Clinical History” (Appendix 2) are described below, which comprise, together with the Clinical Examination, the “MMBGR Protocol – Infants and Preschoolers”.

**Table 3:** Percentage of agreement among experts and the Content Validity Index (CVI) regarding specific data from the Instructive and Clinical History of the MMBGR Protocol

**The number of appraisers according to**	**Nº of Items (%)**	**CVI (%)**	**p-value**
**Instructive**			
10	0 (0,0)	100	1,000
9	3 (30,0)	90	0,972
8	3 (30,0)	80	0,851
7	1 (10,0)	70	0,617
6	2 (20,0)	60	0,350
5	0 (0,0)	50	0,150
4	1 (10,0)	40	0,047
**Clinical history**			
10	26 (40,6)	100	1,000
9	15 (23,4)	90	0,972
8	13 (20,3)	80	0,851
7	8 (12,5)	70	0,617
6	1 (1,6)	60	0,350
5	1 (1,6)	50	0,150

Binomial Exact Test

**Caption:** CVI = Content Validity Index; % = percent

The “**instructive**” includes guidelines on the use of the MMBGR Protocol – Infants and Preschoolers, both in relation to the application and to the Registration in the Protocols of “Orofacial Myofunctional Clinical History” (anamnesis/interview) and “Clinical Examination” (Orofacial Myofunctional Exam with Scores). It also explains the main objective of the MMBGR, aimed at the speech therapist to assess, diagnose, and establish a prognosis in OM; the average time for its application (30-45 minutes for “Clinical History”, and 60-90 minutes for the “Clinical Examination”), with a demand of about 2 hours of work for analysis of the data obtained.

The instructive also provides information about the Clinical Examination: I. Procedures for the collection/analysis of the Orofacial Myofunctional Examination, including photograph/video recording standards, static and dynamic image recording scripts; II. Procedures on items to be evaluated, age groups and scores; III. General guidelines on aspects of the protocol to be considered, according to the age group, including material used and form of recording the following items:

Identification and anthropometric data, followed by the items of the orofacial myofunctional exam: **extraoral exam** – subjective facial analysis, lips and jaw; **intraoral examination** – lips, cheeks, tongue, palate, palatine tonsils, teeth and occlusion; **tone** – lips, mentum, tongue and cheeks; **orofacial functions** – Breathing, Sucking, Chewing, Swallowing (liquid, pasty and solid/semi-solid) and Speech.

In the anamnesis/initial interview situation, the Protocol for Surveying the "Clinical History" includes aspects to be raised with the person responsible for the infant/preschooler. Most items can be checked if there is an occurrence, and there are spaces for complementing information, descriptions and observations. It includes information ranging from the identification of the infant/preschooler (registration number, name, dates (exam and birth), age, informant, education, address, family and contact information) to data directly related to the complaint and the reason for referral to Speech Therapy.

The developmental aspects are organized by occurrence and period, as well as recording the difficulties encountered; a referential chronology on the acquisition of each behavior is included. There are items addressing aspects of development and motor difficulty, feeding (from breastfeeding to the use of utensils), as well as health, respiratory, and sleep problems; speech therapy treatments and in interdisciplinary areas; and the occurrence of harmful habits. The current feeding, chewing, swallowing, speech, oral communication, and voice patterns of the infant or preschooler are also listed.

## DISCUSSION

The study was designed to adapt and validate the content and appearance of the MBGR Orofacial Myofunctional Assessment Protocol for use in infants and preschoolers. Thereunto, the Instructive was developed based on the authors' professional experience and the bibliographic references consulted, and versions of the "Clinical History Protocols" and "Orofacial Myofunctional Clinical Examination" were developed with scores to be used with patients aged 6 months to 5 years and 11 months to live.

We consider elaborating the Instructive so that it functions as an instructional guide that guides the speech therapist's use of the protocol, standardization of records, and documentation. The instructive guide is an integral part of the MMBGR Protocol – Infants and Preschoolers, and its application must comprise the clinical reasoning inherent to the application of the Clinical History and Clinical Examination protocols.

Only the Instructive and the Protocol Clinical Myofunctional Orofacial History were presented in this article. The analysis of the protocol's content allowed for the retention, addition, and deletion of items, and, given the scarcity of validated protocols in the OM area for children under the age of six, the main aspects addressed were drawn from the reference literature on child development.

The MMBGR Clinical History protocol now includes data on siblings, as family constitution is an important aspect to understand in the therapeutic process^(4)^. Items that did not correspond to the age group, on the other hand, were excluded. Aspects of global motor development were also included, as the progression of body control evolves into a set of acquisitions, balance in different postures and positions, and functional independence for the child^(17)^, which is fundamental for the feeding situation. The motor patterns addressed and which motor skills changed were based on the Alberta Infant Motor Scale (AIMS): Reference values for categorizing children's motor performance^(17,18)^.

Aspects of feeding development, with standards by age group, were based on the Food Guide for Brazilian children under 2 years old, published by the Ministry of Health of Brazil^(19)^, and on international protocols^(1,3)^, since there are no instruments validated in Brazil for this age group. The ChOMPS - *Child Oral and Motor Proficiency Scale protocol* ^(1)^ was used because it is an instrument that investigates aspects of eating, drinking, and eating skills based on parental reports, such as the ability to bite soft food and drink thin liquids without coughing or choking, as well as motor skills that support safe swallowing and independent eating, such as the ability to bite soft food and drink thin liquids without coughing or choking^(1)^.

Given the importance of detecting eating difficulties early, the aspects addressed by Thoyre *et al*. (2018) in accordance with the Pediatric Eating Assessment Tool - PediEAT protocol^(3)^, which assesses symptoms of eating problems, seek to identify eating problems early, based on parental report. Research was considered^(20,21)^ whose reports of mothers refer, among other aspects, to the difficulty of introducing utensils, to the baby's ability to drink from a cup (with and without a lid), to drinking it with a straw (large and small), and whether the child has autonomy or is assisted by the mother when using the utensil to drink. Similar data on spoons (type and use) were considered in this adaptation. Breastfeeding is generally recommended for up to 6 months of life, with continuation until 24 months, as a supplement to the diet, which prioritizes the introduction of new consistencies that promote the development of the stomatognathic system.

Studies on the development of speech were used to inform aspects of communication and speech ^(22,23)^, focusing solely on articulatory production aspects related to OM at early ages.

We believe that the obtained agreement values are positive, which is consistent with other studies that used the CVI to analyze the content and appearance of instruments in the OM area^(11)^. We emphasize that, following the second analysis, 100 percent of the experts said they “completely agree” with the new instrument's content and appearance. The Orofacial Myofunctional Clinical History instructive and protocol, which are part of the MMBGR Protocol – Infants and Preschoolers, fill an important gap in the OM clinic's and research's knowledge of speech-language pathology. For the remaining stages of the validation process, new studies must be proposed. To enable applicability in the population in question, our research group has been working on the subsequent step of “validity evidence based on response processes,” as recommended in the literature^(7)^.

## CONCLUSION

The MBGR Orofacial Myofunctional Assessment Protocol was adapted for use in infants and preschoolers, and the Orofacial Myofunctional Clinical History Instructive and Protocol was validated in terms of content and appearance, allowing it to be used for age groups ranging from 6 months to 5 years and 11 months of life.

## Appendix 1 MMBGR Protocol – infants and preschoolers – Instructive

MMBGR PROTOCOL INFANTS AND PRESCHOOLERS

Andréa Monteiro Correia Medeiros, Irene Queiroz Marchesan, Katia Flores Genaro, Giédre Berretin-Felix

Instructive of application and registration

The MMBGR Protocol – Infants and Preschoolers is an instrument in the field of Orofacial Motricity with scores, indicated to be used in the population from 6 months to 5 years and 11 months of life.

It consists of the CLINICAL HISTORY (history/interview) and OROFACIAL MYOFUNCTIONAL EXAM WITH SCORES (evaluation) protocols, intended for the speech therapist to assess, diagnose and establish a prognosis in Orofacial Motricity.

To apply it, an average time of 30-45 minutes is spent for CLINICAL HISTORY, and 60-90 minutes for data collection from the CLINICAL EXAM, and the analysis of the results requires about 2 hours of work.

Some domains can be analyzed through the Clinical History and others through the Clinical Examination, while clinical reasoning is performed by understanding the relationship between the data recorded in the two protocols.

CLINICAL HISTORY PROTOCOL:

It is applied with the person responsible (main caregiver) for the infant/preschooler, before performing the orofacial myofunctional exam.

A private and peaceful environment is chosen, with the data collected directly by the speech therapist and registered at the time of the survey, with the person in charge.

It contains items that include: identification, complaints, family history and complications; development and motor difficulties; general and/or specific health problems, such as breathing, sleep and treatments performed; aspects related to feeding from breastfeeding to current feeding, including main difficulties and feeding pattern; as well as about breathing, chewing, swallowing, oral habits and also aspects about speech, oral communication, hearing and voice.

OROFACIAL MYOFUNCTIONAL EXAM WITH SCORES PROTOCOL:

It is applied directly to the infant/preschooler, preferably with the person responsible in the room during the exam, especially in subjects aged up to 23 months of age. It is also considered that, depending on the age and level of understanding of the child, the questions should be addressed to them, in a language suitable for their understanding and obtaining an answer, with the agreement of the person responsible.

A private and quiet environment is chosen, with good lighting, and all data can be collected directly by the speech therapist with the subject and duly registered in the protocol at the time of the survey (real time).

Registration of the exam through photographic and video documentation, for later certification and analysis of previously collected data, is also recommended.

I. Positioning procedures in the Orofacial Myofunctional Examination and documentation standardization:

Both in real-time observation and during the analysis of the photograph and/or video record, the subject should be observed sitting facing the examiner, with the back supported, with head correction:

▪ Infant (6 to 23 months): on the guardian's lap;

▪ Preschool (24 to 71 months): in a chair, keeping the feet in contact with the floor (chair suitable for the subject's size). Up to 47 months, it can be placed on the guardian's lap.

A standard size chair can be used, and in these cases, when the patient's height does not allow for plantar support, use a footrest that guarantees an angle equivalent to the smaller chair.

The examiner should be seated, facing the subject, keeping their eyes level with the infant/preschooler's eyes. Positioning indications must include an appropriate ergonomic shape for the examination for both (examiner and subject).

For recording in photography/video, some standardizations must be observed:

▪ The distance between the camera tripod and the subject must be the same in all assessments. There may even be signs on the floor and walls as to the planes and angles to be standardized for the subject's positioning, when recording the images.

The distances for each photographic and video record may vary according to the evaluator's needs, as well as the specifics of each equipment used (camera, flash and lenses), the physical space and the light in the room. (Frazão, Manzi, 2019)

To document the intraoral region, the distances should be closer between the tripod and the subject than for the face registration. It is recommended to use macro lenses on the camera. (Frazão, Manzi, 2019).

The Protocol presents the following Recommended Roadmap for Image Registration. However, depending on the evaluator's needs, as well as on the subject's age, ability to understand and execute the movement, other images can be registered (extra images).

Reference: Frazão YS, Manzi SHB. Atualização em documentação fotográfica e em vídeo na motricidade orofacial. In: Silva HJ da, Tessitore A, Motta AR, Cunha DA da, Berretin-Felix G, Marchesan IQ, editors. Tratado de Motricidade Orofacial. 1st ed. São José dos Campos: Pulso Editorial; 2019. p. 243–53.

**Table:**

Script for registration of images					
	Static Images				
	- Face:	[ ] Frontal view without head posture correction		[ ] Front view with corrected head posture	
	- Lips:	[ ] At rest - usual	[ ] Internal mucosa	[ ] Superior labial frenulum	
	- Cheeks:	[ ] Right internal mucosa	[ ] Left internal mucosa		
	- Tongue:	[ ] Externalized *(out of the oral cavity)*			
		[ ] Frenulum *(tongue raised without touching the palate)*		[ ] Frenulum *(high tongue with maneuver)*	
	- Palate:	[ ] Hard			
	- Teeth:	[ ] Upper arcade	[ ] Lower archway		
	- Occlusion:	[ ] Anterior	[ ] Right Side	[ ] Left Side	
	- Others:	[ ] At the discretion of the examiner			
			
	**Dynamic Images**				
	- Suction:	[ ] Nourish *(breast)*	[ ] Bottle		
	- Chewing:	[ ] Open mouth after chewing and before swallowing			
	- Swallowing:	[ ] Liquid	[ ] Pasty	[ ] Solid/Semi-solid	[ ] Open mouth after swallowing *(residual)*
	- Speech:	[ ] Semi-directed	[ ] Figure naming/repetition		
	- Oropharynx:	[ ] Soft palate	[ ] Uvula	[ ] Palatine Tonsils	

II. Procedures on items to be evaluated, age groups and scores:

The Infant/Preschool MMBGR Protocol covers identification items and anthropometric data, followed by the items of the orofacial myofunctional exam: **extraoral exam** – subjective facial analysis, lips and jaw; **intraoral examination** – lips, cheeks, tongue, palate, palatine tonsils, teeth and occlusion; **tone** – lips, mentum, tongue and cheeks; **orofacial functions** – Breathing, Sucking, Chewing, Swallowing (liquid, pasty and solid/semi-solid) and Speech.

Most items are obtained for all age groups, while others follow the age range indicated in the instrument itself. This is because the entire instrument was developed respecting the expected development, and possible to be carried out, in each age group addressed. However, each item can be applied beyond or below the suggested ages, according to the child's individual development.

For analysis purposes, scores are assigned by age group in months for each item evaluated, and at the end, the scores must be registered in the Summary of Orofacial Myofunctional Exam. Higher scores are related to the worst patterns of orofacial motricity observed in infants and preschoolers. The best and worst possible results to be obtained by age group are described in the summary table. However, these values ​​should not be adopted as a diagnostic cutoff score, but can serve as individual reference values ​​during the follow-up/rehabilitation process of each patient.

III. General guidelines on aspects of the protocol to be considered, according to age group:

**Table:**

**1. Identification**
✓All age groups. ✓Data obtained directly from the guardian, and/or extracted from the child's health booklet.
**2. Extraoral Exam**
✓All age groups. ✓On-site assessment and/or image recording (photo) in front view, for analysis after the orofacial myofunctional exam.
**3. Intraoral Exam**
✓All age groups *(differentiated scores for infants and preschoolers, depending on the item evaluated)* ✓On-site assessment and/or image recording (photo) with a macro lens, for analysis after the orofacial myofunctional exam. ✓Examiner should perform the intraoral inspection of the subject, wearing procedural gloves, as in the examination of the frenulum of the tongue; using lip retractor for evaluation of teeth and occlusion *(when there is difficulty in placing the lip retractor in children from six months to four years of age, it is requested that the lips be parted with the guardian's fingers).* ✓The examiner should also ask the child to open their mouth voluntarily and/or by imitation, to observe structures such as tongue, palate and palatine tonsils.
**4. Tone**
✓All age groups. ✓The examiner must obligatorily perform visual observation and palpation of the structures. ✓The impression of the result, from the palpation exam must be registered in real time, and later confirmed, when possible, by the image taken.
**5. Orofacial Functions**
✓For each orofacial function related to feeding, always use the same type of food, observing its consistency. Exceptions can be made regarding the type of food, according to the subject's habit and individual acceptance/rejection. ✓It is always recommended to use the food brought by the family. ✓ In speech evaluation, for picture naming, it is recommended to use the material belonging to the instrument *(MMBGR Protocol - Figures for Naming).* ✓ Video recording *(footage)* It is recommended to be used in the analysis after the orofacial myofunctional exam.

**Table:**

General guidelines on aspects to be documented/evaluated: Orofacial Functions			
**Orofacial Function**	**Age group**	**Material used**	**Exam/ Specific Registration**
Breathing	▪All (6 to 71 months)	▪ Glatzel Mirror/ ▪ Altmann's Millimeter Nasal Mirror	▪Measure airflow in real time ▪It is not mandatory to clean with saline solution, but it is recommended that the subject blows their nose before the procedure. ▪Mark airflow on graph paper
Suction/Swallowing	▪Up to 23 months, as long as you are still breastfed	▪Breast *(breast milk)*	▪Observe breast feeding *(2 to 5 minutes)*
Suction/Swallowing	▪Up to 23 months, as long as you still use a bottle in your routine	▪Bottle *(breast milk or milk formula)*	▪Observe bottle offer *(3 minutes or at least 10 ml)*
Chewing	▪From 12 months	▪Hand/cutlery *(French bread or biscuit)*	▪Observe for 2 minutes, or two complete cycles, until swallowing occurs. ▪From 36 months on, ask the subject to open their mouth before swallowing, to check the efficiency of the food crushing.
Swallowing - liquid	▪From 12 months	▪Cup *(water, juice, or breast milk/formula)*	▪Observe emptying of at least ¼ cup (50 ml)*.*
Swallowing - pasty	▪Up to 11 months ▪Up to 23 months, when they feed in a pasty consistency	▪Spoon *(porridge, puree, mashed food such as banana)*	▪Observe for 2 minutes, or a complete cycle, until swallowing is complete *(laryngeal elevation)*
Swallowing - solid/semi-solid	▪From 12 months	▪Hand/Cutlery *(French bread, biscuits, among others)*	▪Observe for 2 minutes, or a complete cycle, until swallowing is complete *(laryngeal elevation)* ▪Ask the subject to open their mouth after swallowing to check for food residue.
Speech - Production of phones and phonemes	▪From 24 months onwards	▪List of Figures *(MMBGR Protocol - Figures for Naming)* ▪Semi-directed speech	▪Figure Naming: the subject has to say the name of the figure shown. ▪If you don't know, the examiner names the figure, and after showing another figure in the list, it shows the figure not named initially. The subject is then asked to name it. ▪ After that, you will not be given another chance. It is only registered that the subject was unable to produce even with the examiner's attempt to repeat. ▪Semi-directed speech: it can be observed throughout the orofacial myofunctional exam and also by asking the subject to say their name and age; talk about school or a joke; talk about a trip or tour ▪Elaboration of the phonemic picture, based on the subject's production.
Speech - General Aspects of Phonoarticulation	▪From 36 months onwards	▪Semi-directed speech	▪Semi-directed speech: can be observed throughout the orofacial myofunctional exam and also by asking the subject to say their name and age; talk about school or a joke; talk about a trip or tour

## Appendix 2 MMBGR protocol - Infants and preschools: clinical myofunctional orofacial history

MMBGR PROTOCOL **-** INFANTS AND PRESCHOOLS (6 months to 5 years and 11 months)

Andréa Monteiro Correia Medeiros, Irene Queiroz Marchesan, Katia Flores Genaro, Giédre Berretin-Felix

**Table:**

**Name**: ___________________________________________		**Nº**: ________________
**Exam date**: ___ / ___ / ___	**Age:** _____ years and _____ months	**BD:** ____ / ____ / ____
**Informer:** _______________________	**Degree of kinship:** ________________________________________	

**Table:**

**Attend daycare/school:** ❒ no ❒ yes	**Class:** _________	**Period:**❒ morning ❒ afternon ❒ full-time

**Table:**

**Address:** ________________________________________________	**Nº:** ________	**Complement:** ______________
**Neighborhood:** ___________________________	**City/State:** __________________	**Zip-code:**__________________

**Table:**

**Affiliation:**	Father's name: __________________________________		Mother’s name: ____________________________
	E-mail: _______________________________________		E-mail: __________________________________
	Phone number: ( ___ ) ___________________________		Phone number: ( ___ ) ______________________
	Work phone number: ( ___ ) ______________________		Work phone number: ( ___ ) __________________
**Siblings:**	❒ no	❒ yes – How many: _____________________	Sibling’s age: _________________________

**Table:**

**Who recommended for Speech Therapy?** *(Name, specialty, phone number):* *____________________________________________________________________________________________________*
**For what reason?** _____________________________________________________________________________________

**Table:**

**Main complaint (of the responsible):** _____________________________________________________________________

**Table:**

**Other related complaints:**						
❒ lips	❒ teeth	❒ lingual frenulum	❒ suction	❒ chewing	❒ speech	❒ body posture
❒ tongue	❒ occlusion	❒ swallowing	❒ breathing	❒ voice	❒ hearing	

Additional Information: ____________________________________________________________________________________________

**Table:**

**Family history (in relation to the complaint):**	
❒ no	❒ yes – What complaint: ________________________________________________________________________

**Table:**

**Complications:**		
In pregnancy:	❒ no	❒ yes – What complaint: ____________________________________________________
At birth:	❒ no	❒ yes – What complaint: ____________________________________________________

**Table:**

**Motor development** (*check if you have already performed the behavior and at what age*)**:**				Do not know/ Do not remember		Age expected
Hold the head	❒ no	❒ ye**s**: ___ months		❒		3 months
First attempts at locomotion: dragging, crawling	❒ no	❒ ye**s**: ___ months		❒		6 to 7 months
Sit independently	❒ no	❒ ye**s**: ___ months		❒		7 months
Play in lateral decubitus	❒ no	❒ ye**s**: ___ months		❒		7 months
Roll	❒ no	❒ ye**s**: ___ months		❒		7 months
Sit without support (uses hands: exploratory activity)	❒ no	❒ ye**s**: ___ months		❒		8 months
Sit firmly (manipulates and explores objects with hands)	❒ no	❒ ye**s**: ___ months		❒		9 months
Crawl	❒ no	❒ ye**s**: ___ months		❒		10 months
Stand up	❒ no	❒ ye**s**: ___ months		❒		10 months
Walk without support	❒ no	❒ ye**s**: ___ months		❒		13 months
In general, motor development can be considered	❒ normal	❒ altered				
If changed, tick which skills:	[ ] hold the head		[ ] sit		[ ] stand up	
	[ ] roll		[ ] crawl		[ ] walk without support	

**Table:**

**Motor difficulty:**	❒ no	❒ yes:	[ ] run	[ ] riding a tricycle/bike with support wheels	[ ] dress up
			[ ] tie the shoes	[ ] button up	[ ] brushing teeth
			[ ] paint (hold pencil)	[ ] Use spoon/fork	[ ] others: _________________

**Table:** Health problems

			**Describe**	**Treatment/Medication**
Neurological:	❒ no	❒ yes:	_____________________	_______________________________________
Orthopedic:	❒ no	❒ yes:	_____________________	________________________________________
Metabolic:	❒ no	❒ yes:	_____________________	________________________________________
Digestive:	❒ no	❒ yes:	_____________________	________________________________________
Hormonal:	❒ no	❒ yes:	_____________________	________________________________________
Otorhinolaryngologic:	❒ no	❒ yes:	_____________________	________________________________________
Pneumological:	❒ no	❒ yes:	_____________________	________________________________________
Cardiac:	❒ no	❒ yes:	_____________________	________________________________________
Emotional/Psychic:	❒ no	❒ yes:	_____________________	________________________________________

Other problems: ________________________________________________________________________________________________________________

**Table:** Breathing problems

			**Treatment/Medication**
Frequent colds*****:	❒ no	❒ yes:	_________________________________________________________
Throat Problems:	❒ no	❒ yes:	________________________________________________________________
Tonsillitis:	❒ no	❒ yes:	________________________________________________________________
Halitosis:	❒ no	❒ yes:	________________________________________________________________
Asthma:	❒ no	❒ yes:	________________________________________________________________
Bronchitis:	❒ no	❒ yes:	________________________________________________________________
Pneumonia:	❒ no	❒ yes:	________________________________________________________________
Rhinitis:	❒ no	❒ yes:	________________________________________________________________
Sinusitis:	❒ no	❒ yes:	________________________________________________________________
Nasal obstruction:	❒ no	❒ yes:	________________________________________________________________
Itchy nose:	❒ no	❒ yes:	________________________________________________________________
Coryza:	❒ no	❒ yes:	________________________________________________________________
Sneezing in salute:	❒ no	❒ yes:	________________________________________________________________

* *Colds on a regular basis (changes in upper airway) up to 5 years of age: more than 12 episodes per year*

Other problems: _____________________________________________________________________________________________

**Table:** Sleep

Bedtime: _____	Wake up time: _____		
Nocturnal: ❒< 8 hours	❒> 8 hours: describe the frequency ____________________________________		
Sleeps during the day:	❒ no	❒ yes:	_______________________________________________
Hectic:	❒ no	❒ yes:	_______________________________________________
Fragmented:	❒ no	❒ yes:	_______________________________________________
Nocturnal Enuresis:	❒ no	❒ yes:	_______________________________________________
Snoring:	❒ no	❒ yes:	_______________________________________________
Snores:	❒ no	❒ yes:	_______________________________________________
Sialorrhea:	❒ no	❒ yes:	_______________________________________________
Apnea:	❒ no	❒ yes:	_______________________________________________
Bruxism:	❒ no	❒ yes:	_______________________________________________
Water intake at night:	❒ no	❒ yes:	_______________________________________________
Open mouth when sleeping:	❒ no	❒ yes:	_______________________________________________
Dry mouth on waking up:	❒ no	❒ yes:	_______________________________________________
Hand placed under face:	❒ no	❒ yes: [ ] R [ ] L _______________________________________________	
Posture: ❒ lateral decubitus	❒ supine		❒ ventral decubitus

Other problems: ___________________________________________________________________________________________________________________

**Table:** Treatments

				**Name/Contact**
**Speech therapy:**	❒ no	❒ in the past	❒ nowadays	
Reason: ________________________________________				___________________________
**Medical:**	❒ no	❒ in the past	❒ nowadays	
which specialties: _________________________				
Reason: ________________________________________				___________________________
**Psychological:**	❒ no	❒ in the past	❒ nowadays	
Reason: ________________________________________				__________________________
**Physiotherapy:**	❒ no	❒ in the past	❒ nowadays	
Reason: ________________________________________				__________________________
**Dental:**	❒ no	❒ in the past	❒ nowadays	
Reason: ________________________________________				__________________________
Type of Procedure: ____________________________				
**Surgical:**	❒ no	❒ in the past	When? _______	
Reason: ________________________________________				__________________________
Type of Procedure: ____________________________				

Other treatments: _______________________________________________________________________________________________

**Table:** Feeding Development Breastfeeding

Breast (Breastfeeding):	❒ no	❒ yes	
Exclusive:	❒ no	❒ yes - Until when: _______________	❒ don't know/don't remember
Complemented:	❒ no	❒ yes - Until when: _______________	❒ don't know/don't remember

**Table:** Feeding - difficulty in introducing

Utensils:	❒ straw	❒ cup	❒ spoon	❒ fork	Describe: _____________________
Flavors:	❒ salty	❒ sweet	❒ sour	❒ bitter	Describe: _____________________
Consistency:	❒ liquid	❒ pasty	❒ semi-solid	❒ solid	Describe: _____________________

**Table:** **Utensil currently used to provide food** (you can check more than one alternative)**:**

❒ bottle – beak type: _______________ material: _______________ hole: _______________			
❒ regular cup (open)	❒ cup with lid	❒ cup with valve	❒ cup with straw
❒ spoon	❒ fork		❒ other ____________________

**Table:** Current feeding pattern

**Ready behavior and feeding pattern**			**Do not know/** **Do not remember**	**Expected** **Age**
Open mouth towards the spoon when hungry	❒ no	❒ yes	❒	6 months
Leans forward towards the spoon, holding the hand of the person offering the food	❒ no	❒ yes	❒	6 months
Accepts food scraped and/or crushed with a spoon	❒ no	❒ yes	❒	6 to 7 months
Leans to the spoon, picks up or points the food	❒ no	❒ yes	❒	7 to 11 months
Accepts food mashed with a fork, containing small pieces	❒ no	❒ yes	❒	7 to 8 months
Accepts chopped or small pieces of food	❒ no	❒ yes	❒	9 to 11 months
Eats independently but still needs help	❒ no	❒ yes	❒	9 to 12 months
Use/combine words and gestures to express the desire for food	❒ no	❒ yes	❒	12 to 24 months
Accepts food in adult consistency	❒ no	❒ yes	❒	from 12 months
Eat independently with spoon	❒ no	❒ yes	❒	12 to 24 months
Hold the cup with both hands	❒ no	❒ yes	❒	12 to 24 months
Demonstrates ability to chew	❒ no	❒ yes	❒	12 to 24 months

**Table:** Current feeding

**Type**		Describe frequency	Which?
Fruits:	❒no	❒ yes: _______________	___________________________________
Vegetables:	❒no	❒ yes: _______________	___________________________________
Leguminous:	❒no	❒ yes: _______________	___________________________________
Cereals: *(rice, pasta, wheat)*	❒no	❒ yes: _______________	___________________________________
Grains: *(beans, lentils, peas)*	❒no	❒ yes: _______________	___________________________________
Meet:	❒no	❒ yes: _______________	___________________________________
Milk and derivatives:	❒no	❒ yes: _______________	___________________________________
Sugars:	❒no	❒ yes: _______________	___________________________________

**Table:** In general, eat predominantly:

Subject preference:	❒ liquids	❒ pasties	❒ solids	❒ different consistencies
Offered by the caregiver/guardian:	❒ liquids	❒ pasties	❒ solids	❒ different consistencies

**Table:** Most of the time, with whom, how and where you eat:

With which company:	❒ alone	❒ family	❒ colleagues (*school*)		❒ other - which: ________________
no other activity:	❒ at the table	❒ couch	❒ in floor	❒ in bed	❒ other - which: ________________
Watching TV:	❒ at the table	❒ couch	❒ in floor	❒ in bed	❒ other - which: ________________
Joking:	❒ at the table	❒ couch	❒ in floor	❒ in bed	❒ other - which: ________________
Using tablet/cell phone:	❒ at the table	❒ couch	❒ in floor	❒ in bed	❒ other - which: ________________
Carrying out other activities:	❒ at the table	❒ couch	❒ in floor	❒ in bed	❒ other - which: ________________

**Table:** Chewing

Lips:	❒ closed	❒ ajar	❒ open		
Noise:	❒ no	❒ sometimes	❒ yes		
Pain or discomfort during chewing:			❒ no	❒ sometimes	❒ yes - [ ] right [ ] left
Possible causes of pain when chewing: ________________________________________________________________________________					
Chewing difficulty:		❒ no	❒ yes - What: ________________________________________________________		
Escaping food while chewing:			❒ no	❒ yes	
Need to drink liquid with food:			❒ no	❒ yes	

Other problems: _____________________________________________________________________________________________________

**Table:** Chew the foods:

	**properly**	**little**	**very**	**Does not know how to inform know/did not observe**
Compared to family:	❒	❒	❒	❒
Compared to friends:	❒	❒	❒	❒
Describe: ____________________________________________________________________________________________				

**Table:** Chewing speed

	**similar**	**faster**	**slower**	**Does not know how to inform know/did not observe**
Compared to family:	❒	❒	❒	❒
Compared to friends:	❒	❒	❒	❒
Descrever: ____________________________________________________________________________				

**Table:** Chewing ability *(degree of satisfaction of the patient's family with regard to chewing)*

❒ excellent	❒ good	❒ regular	❒ bad

**Table:** Swallowing

			Describe frequency
Noise:	❒ no	❒ yes	___________________________________________________________________________
Chokes:	❒ no	❒ yes	________________________________________________________
Pain on swallowing:	❒ no	❒ yes	________________________________________________________
Nasal reflux:	❒ no	❒ yes	________________________________________________________
Previous escape:	❒ no	❒ yes	________________________________________________________
Throat clear:	❒ no	❒ yes	_________________________________________________________
Cough:	❒ no	❒ yes	_________________________________________________________
Residues after swallowing:	❒ no	❒ yes	________________________________________________________
Other difficulties:	❒ no	❒ yes	_________________________________________________________

Other problems: ____________________________________________________________________________________________________

**Table:** Oral habits

Pacifier suction:	❒ no	❒ yes - until when: _______	[ ] common	[ ] orthodontic	[ ] does not know how to inform
Finger Suction:	❒ no	❒ yes - until when: _______			
Tongue suction:	❒ no	❒ yes - until when: _______			
Moisten lips:	❒ no	❒ yes - age: ___________			
Others: _________________________________________			until when: ____________________________		

**Table:** Biting habits

Bruxism *(grit your teeth)*:	❒ no	❒ yes - [ ] daytime	
Teeth clenching:	❒ no	❒ yes - until when: _______	
Onychophagy *(nail biting)*:	❒ no	❒ yes - until when: _______	
Biting/Nibbling lips:	❒ no	❒ yes - until when: _______	
Biting/Nibbling the inner mucosa of the cheeks:		❒ no	❒ yes - until when: _____________________
Biting objects: Which one? _____________________		❒ no	❒ yes - until when: _____________________

**Table:** Posture habits

Lower lip interposition:	❒ no	❒ yes
Tongue interposition:	❒ no	❒ yes
Jaw protraction:	❒ no	❒ yes
Jaw support in hand:	❒ no	❒ yes [ ] right [ ] left
Head support in hand:	❒ no	❒ yes [ ] right [ ] left

Other habits: _______________________________________________________________________________________________________

**Table:** Oral communication

Impaired intentionality:	❒ no	❒ yes - detail: _____________________________________	
Absence of sound production:	❒ no	❒ yes - detail: _____________________________________	
Difficulty understanding:	❒ no	❒ yes - detail: _____________________________________	
It took me to start talking:	❒ no	❒ yes - detail: _____________________________________	
Difficulty in writing sentences:		❒ no	❒ yes - detail: _____________________________
Haw *(hesitation/repeat/extension)*:		❒ no	❒ yes - detail: _____________________________

OBS: In the case of a suspected alteration, apply a specific language protocol

Other problems: ____________________________________________________________________________________________________________________________________________________________________________________________________________________________________________________________________________________________________________________________________________________________

**Table:** Speech

Omission:	❒ no	❒ yes - describe the frequency: _____________________________________________________		
Substitution:	❒ no	❒ yes - describe the frequency: _____________________________________________________________		
Distortion:	❒ no	❒ yes - describe the frequency: _____________________________________________________________		
Excessive salivation:		❒ no	❒ yes - describe the frequency: _________________________________________	
Tongue interposition:		❒ no	❒ yes - [ ] anterior [ ] lateral	
Opening the mouth:		❒ normal	❒ restricted	❒ exaggerated
Impaired intelligibility: ❒ no			❒ yes - describe the frequency: _________________________________________	
which phonemes: __________________________________________________________________________________________________				

Other problems: __________________________________________________________________________________________________________________________________________________________________________________________________________________________________________

**Table:** Hearing

Hypoacusis *(hearing loss)*:	❒ no	❒ sometimes	❒ yes [ ] right [ ] left
Otitis *(ear infection)*:	❒ no	❒ sometimes	❒ yes [ ] right [ ] left
otalgia *(earache)*:	❒ no	❒ sometimes	❒ yes [ ] right [ ] left
Previous audiological evaluation:	❒ no	❒ yes - When: _________________________________________________	
Results: _______________________________________________________________________________________________________			

Other problems: __________________________________________________________________________________________________________________________________________________________________________________________________________________________________________

**Table:** Voice

Hoarseness:	❒ no	❒ sometimes	❒ yes
Weakness:	❒ no	❒ sometimes	❒ yes
Aphonia:	❒ no	❒ sometimes	❒ yes
Hypernasality:	❒ no	❒ sometimes	❒ yes
Hyponasality:	❒ no	❒ sometimes	❒ yes
Vocal abuse:	❒ no	❒ sometimes	❒ yes
Detail: _____________________________________________________________________________			

OBS: In the case of a suspected alteration, apply specific vocal protocol

Other problems: __________________________________________________________________________________________________________________________________________________________________________________________________________________________________________

Additional information:

______________________________________________________________________________________________________________________________________________________________________________________________________________________________________________________________________________________________________________________________________________________________________________________________________________________________________________________________________________________________________________________________________________________________________________________________________________________________________________________________________________________________________________________________

**Table:**

**Speech therapist**: _____________________________________________	**STRCª**:___________________________
