# ERRATUM: MMBGR Protocol - infants and preschoolers: instructive and orofacial myofunctional clinical history

**DOI:** 10.1590/2317-1782/20212022159en

**Published:** 2023-01-06

Due to author's honest mistake the article “MMBGR Protocol - infants and preschoolers: instructive and orofacial myofunctional clinical history” (DOI https://doi.org/10.1590/2317-1782/20212020324), published in CoDAS 2022;34(2):e20200324, was published with errors.

**On Portuguese title, where the text reads:**

Protocolo MMBRG – lactentes e pré-escolares: instrutivo e história clínica miofuncional orofacial

**It should read:**

Protocolo MMBGR – lactentes e pré-escolares: instrutivo e história clínica miofuncional orofacial

**On English version on pages 4, 8, 9 and 12, where the text reads:**

Pasty

**It should read:**

Pudding

The authors apologize for the errors.

