# Supplementary material for: MMBGR Protocol - infants and preschoolers: Instructive and Orofacial Myofunctional Clinical History
Source: Codas. 2022 Jan 10;34(2):e20200324. doi: 10.1590/2317-1782/20212020324 (PMC9851188; doi:10.1590/2317-1782/20212020324)
Supplement: Translation

# Translation: Protocolo MMBRG – Lactentes e Pré-escolares: Instrutivo e História Clínica Miofuncional Orofacial

**Authors:** Andréa Monteiro Correia Medeiros, Irene Queiroz Marchesan, Katia Flores Genaro, Íkaro Daniel de Carvalho Barreto, Giédre Berretin-Felix

**Affiliations:** 1 Departamento de Fonoaudiologia, Universidade Federal de Sergipe – UFS - São Cristóvão (SE), Brasil.; 2 CEFAC Saúde e Educação - São Paulo (SP), Brasil.; 3 Departamento de Fonoaudiologia, Faculdade de Odontologia de Bauru, Universidade de São Paulo – FOB/USP - Bauru (SP), Brasil.; 4 Departamento de Estatística e Informática, Universidade Federal Rural de Pernambuco – UFRPE - Recife (PE), Brasil.

**Keywords:** Fonoaudiologia, Lactente, Pré-Escolar, Estudos de Validação, Anamnese, Diagnóstico, Terapia Miofuncional, Sistema Estomatognático, Diagnosis, Myofunctional Therapy, Stomatognathic System

## Abstract

**Objetivo:**

Apresentar Instrutivo e Protocolo de História Clínica Miofuncional Orofacial pertencentes ao Protocolo MMBGR - Lactentes e Pré-escolares, incluindo adaptação e validação do conteúdo e aparência destes.

**Método:**

Estudo tipo validação, descritivo e transversal. Adaptação a partir do Protocolo MBGR, fundamentada em estudos teóricos e experiência dos autores. Considerados lactentes entre 6 e 23 meses de vida e pré-escolares entre 24 e 71 meses. Obtido consentimento e consenso da versão adaptada pelos autores (originais e atuais). Analisados aparência e conteúdo do novo instrumento por 10 fonoaudiólogos especialistas em Motricidade Orofacial. Realizadas duas rodadas de análise: Primeira com formulário eletrônico contendo questões dicóticas (sim/não), com espaço para justificar as respostas negativas; calculado Índice de Validade de Conteúdo e Teste Binomial Exato; e Segunda com escala Likert 5 posições.

**Resultados:**

Produzido instrutivo inédito e adaptado o Protocolo de História Clínica, com manutenção de 23 itens pertinentes à faixa etária em questão. Excluídas informações em 7 itens e acrescidas em 8 itens. Inicialmente obteve-se concordâncias de 70% do instrutivo, por pelo menos 70% dos especialistas; e 64% dos itens da História Clínica, por ao menos 90% dos especialistas. Obteve-se na segunda rodada 100% de respostas “Concordo totalmente” dos especialistas.

**Conclusão:**

“Instrutivo” e “História Clínica Miofuncional Orofacial” tiveram validade de conteúdo e aparência concluída, e junto ao “Exame Clínico” integram o “Protocolo MMBGR - Lactentes e Pré-escolares”, com potencial contribuição para atuação clínica e na pesquisa em Motricidade Orofacial na faixa etária de 6 meses a 5 anos e 11 meses de idade.

## INTRODUÇÃO

A compreensão do desenvolvimento dos aspectos miofuncionais orofaciais em idades iniciais pode contribuir, para melhor entendimento do funcionamento normal do sistema estomatognático, e atuação antecipada voltada as suas eventuais alterações. Conforme Descritores da Área de Saúde (DECs) são considerados lactentes a faixa etária de 1 a 24 meses de vida, e pré-escolares entre 2 e 5 anos de idade.

O levantamento da história clínica em Fonoaudiologia é crucial para reconhecer e compreender a problemática que envolve o indivíduo e sua família, ao mesmo tempo que permite elucidar questões específicas sobre o desenvolvimento da Motricidade Orofacial (MO), imprescindíveis para nortear a situação do exame clínico e estabelecer raciocínio clínico para a intervenção terapêutica propriamente dita.

O resgate do desenvolvimento das funções orofaciais da criança pequena, utilizando-se de instrumentos padronizados e validados, parte do relato dos pais mediante inquérito familiar^(1–3)^. A aproximação entre o fonoaudiólogo e a família pode ser feita de forma objetiva ou subjetiva^(4)^, a depender dos pressupostos teóricos que subsidiam a compreensão terapêutica e norteiam a prática clínica do fonoaudiólogo.

O uso de protocolos tem sido apontado como importante ferramenta para registro e acompanhamento do atendimento clínico, mas ainda são escassos na Fonoaudiologia^(5)^, necessitando de mais estudos que proponham a organização de materiais validados com propriedades psicométricas confiáveis^(6)^ para a clínica da MO.

Consideram-se no processo de validação de testes em Fonoaudiologia as etapas: Evidência de validade baseada em conteúdo, consistência interna e relação com outras variáveis; Evidência de validade baseada nos processos de resposta; Confiabilidade/Precisão; Equidade; Acurácia; e Evidência de validade baseada nas consequências do teste^(7)^.

Os principais instrumentos que têm sido utilizados na área de MO são os protocolos Avaliação Miofuncional Orofacial com Escores - AMIOFE^(6,8)^ e o Protocolo MBGR^(9,10)^, ambos voltados à população maior de 6 anos de idade. Existem ainda protocolos destinados à faixa etária neonatal, que abordam-aleitamento materno, prontidão para mamada e MO^(11–14)^. Ressaltam-se para lactentes o SOMA (Schedule for Oral Motor Assessment)^(15)^ sobre habilidade motora oral; e para lactentes e pré-escolares, o PAD-PET^(16)^, que aborda risco para disfagia.

Considerando a escassez de instrumentos nacionais para as faixas etárias de lactentes e pré-escolares brasileiros, surgiu a necessidade de realizar a adaptação e respectiva validação do Protocolo MBGR^(10)^ para ser utilizado na faixa etária de 6 meses a 5 anos e 11 meses de vida, tendo sido escolhido por sua abrangência e especificidade na área de MO, atendendo ao propósito de investigação tanto da História Clínica como do Exame Clínico Miofuncional Orofacial, além da construção do respectivo instrutivo.

O objetivo do presente artigo é apresentar o “Instrutivo” e o Protocolo “História Clínica Miofuncional Orofacial”, que compõem o “Protocolo MMBGR – Lactentes e Pré-Escolares”, evidenciando o processo de adaptação e respectiva validação baseada na análise do conteúdo do teste.

## MÉTODO

Estudo tipo validação, descritivo e transversal, realizado entre maio e dezembro de 2019, integrante de um projeto mais amplo, aprovado pelo Comitê de Ética e Pesquisa em Seres Humanos, da Universidade Federal de Sergipe, sob nº protocolo CAAE 12529419.6.0000.5546. Todos os responsáveis pelos participantes da pesquisa assinaram o Termo de Consentimento Livre e Esclarecido.

Trata-se da validação de um novo instrumento, adaptado a partir do MBGR^(10)^, destinado à população de lactentes e pré-escolares, tendo sido seguidas as diretrizes preconizadas em estudos de validação, considerando-se a etapa baseada no conteúdo do teste^(7)^, após parecer escrito favorável à adaptação do protocolo pelas autoras do protocolo MBGR original.

Inicialmente foi feito o estudo teórico sobre o desenvolvimento miofuncional orofacial e funções estomatognáticas em idades iniciais, por meio de busca nas plataformas Scielo, Pubmed e Bireme, referente ao período de 1993 a 2017. Utilizados os descritores “Fonoaudiologia”, “Lactentes”, “Pré-escolares”, “Métodos de Avaliação” e “Sistema Estomatognático”, considerando-se periódicos e dissertações/teses disponíveis na íntegra; com ênfase em protocolos da área de MO, validados no Brasil. Contou-se com a expertise dos autores, quanto ao conhecimento dos instrumentos existentes.

Foi construído o instrutivo (guia instrucional) e elaborada a versão adaptada da “História Clínica Miofuncional Orofacial”, considerando-se aspectos sintáticos e semânticos, que foram submetidos à apreciação dos autores originais, e após terem sido revisadas as sugestões e obtido consenso entre os autores (versões original e atual), foi realizada a análise da aparência e do conteúdo do novo instrumento.

Dessa etapa participaram 10 fonoaudiólogos especialistas na área de MO, de quatro regiões do Brasil (Centro Oeste, Nordeste, Sudeste e Sul), convidados a participar do estudo por meio de e-mail e/ou aplicativo de mensagens. Foi encaminhada carta explicativa sobre o objetivo do trabalho, análise e tempo estimado para preenchimento de formulário eletrônico, com respectivo link de acesso, o qual continha instruções específicas sobre como avaliar cada item e o instrumento como um todo.

A maioria dos especialistas estavam na faixa etária entre 41 e 50 anos de idade (70%); todos com titulação de pós-graduação (80% Doutorado e 20% Mestrado) e atuação na área de MO, a maioria por mais de 15 anos (90%) e com experiência docente há pelo menos cinco anos, todos eles escolhidos por atuarem com lactentes (80%) e pré-escolares (80%), conforme Tabela 1.

**Tabela 1:** Caracterização sociodemográfica e profissional dos fonoaudiólogos especialistas

	N	%
**Faixa-Etária**		
Entre 31 e 40 anos	3	30,0
Entre 41 e 50 anos	7	70,0
**Atualmente em qual região do país exerce a profissão:**		
Centro Oeste	2	20,0
Nordeste	3	30,0
Sudeste	1	10,0
Sul	4	40,0
**Qual sua titulação?**		
Doutor	8	80,0
Mestre	2	20,0
**Docência. Há quanto tempo?**		
Menos que 5 anos	1	10,0
Entre 5 e 10 anos	3	30,0
Entre 10 e 15 anos	1	10,0
Entre 15 e 20 anos	3	30,0
Entre 20 e 25 anos	2	20,0
**Anos de experiência na Fonoaudiologia – Motricidade Orofacial.**		
Entre 5 e 10 anos	1	10,0
Entre 15 e 20 anos	4	40,0
Entre 20 e 25 anos	5	50,0
**Atua na área de Motricidade Orofacial com Lactentes (até 24 meses de vida)?**	8	80,0
**Há quanto tempo?**		
Menos que 5 anos	1	12,5
Entre 5 e 10 anos	2	25,0
Entre 10 e 15 anos	3	37,5
Entre 15 e 20 anos	2	25,0
**Atua na área de Motricidade Orofacial com pré-escolares (24 meses a 5 anos de vida)?**	8	80,0
**Há quanto tempo?**		
Entre 5 e 10 anos	2	25,0
Entre 10 e 15 anos	1	12,5
Entre 15 e 20 anos	1	12,5
Entre 20 e 25 anos	4	50,0

**Legenda:** n = frequência absoluta; % = Frequência relativa percentual

Como critério de inclusão, o fonoaudiólogo especialista deveria ter: tempo maior que cinco anos de atuação e/ou atividade docente em Fonoaudiologia; ter titulação e/ou publicações relacionadas à área de MO, e expertise na temática, com atuação junto a lactentes e/ou pré-escolares. Em relação aos critérios de exclusão, pareceres incompletos e especialistas que não os entregassem nos prazos estipulados (cerca de 15 dias para análise, de cada etapa).

A primeira apreciação do novo instrumento pelos fonoaudiólogos foi feita por meio de formulário eletrônico contendo questões dicóticas (sim/não), com campos para justificar as respostas negativas. Assim, em caso de não concordância com determinado item, havia espaços disponíveis para descrever sobre qual aspecto não estava de acordo, podendo ainda sugerir algum ajuste.

A análise estatística foi realizada por meio do Índice de Validade de Conteúdo (por item) e teste binomial exato, com nível mínimo de concordância de 70%. Na segunda apreciação de análise do instrumento, pelos mesmos especialistas, foi utilizada a escala de Likert, com cinco opções (concordo plenamente, concordo, indiferente, discordo e discordo totalmente).

## RESULTADOS

Considerou-se pertinente o protocolo MBGR ser adaptado ao público de 6 meses a 5 anos e 11 meses de vida, visto que a sua versão original, destina-se às faixas etárias mais avançadas, indicado para ser aplicado em crianças, adolescentes, adultos e idosos^(10)^. Os resultados aqui apresentados são referentes ao “Instrutivo” e “História Clínica Miofuncional Orofacial” que integram o “Protocolo MMBGR - Lactentes e Pré-escolares”.

O instrutivo foi completamente criado e proposto para compor o novo instrumento denominado “Protocolo MMBGR - Lactentes e Pré-escolares”, em referência à faixa etária alvo, e com o acréscimo da letra M, acrônimo do sobrenome do pesquisador e autor principal dessa versão. Na adaptação do protocolo de “História Clínica Miofuncional Orofacial” do MBGR^(10)^ para lactentes e pré-escolares verificou-se a manutenção de 23 itens pertinentes à faixa etária em questão, foram excluídas informações em 7 itens e acrescidas em 8 itens, que serão descritas a seguir:

Mantidos os itens relacionados aos Dados de identificação; Queixa principal e outras queixas; Antecedentes familiares; Intercorrências; Desenvolvimento e dificuldades motoras; Problemas de saúde; Problemas respiratórios; Sono; Tratamentos; Amamentação; Alimentação – introdução e alimentação atual; Mastigação; Deglutição; Hábitos – orais, de mordida e de postura; Fala; Comunicação; Audição; Voz; e Informações complementares.

Acrescentados itens sobre Dados pessoais - Irmãos (idade dos irmãos); Desenvolvimento Motor (se já realizou essa habilidade motora, com qual idade realizou, além de ter sido inserido no protocolo a informação sobre o comportamento que é esperado por faixa etária), quais habilidades motoras alteradas (sustentar cabeça/rolar/sentar/engatinhar/ficar em pé/andar sem apoio); Tratamentos (ortopedia funcional dos maxilares); Desenvolvimento da alimentação (padrão alimentar e faixa etária); Amamentação (bico); Alimentação (dificuldade em introduzir utensílios); Comunicação (quais sons ausentes).

Excluídos os itens não pertinentes à faixa etária abarcada, tais como: Dados de identificação sobre estado civil, estudo, trabalho, atividade física; Queixas relacionadas à aprendizagem, movimentos mandibulares e dor nos ombros; Frequência da dificuldade motora; Frequência dos problemas respiratórios; Aspectos de tratamento odontológico (implante, exodontia, prótese); Hábitos orais (cigarro, cachimbo); e Escolaridade. As modificações realizadas no Protocolo “História Clínica Miofuncional Orofacial”, estão descritas na Tabela 2.

**Tabela 2:** Itens modificados na História Clínica para constarem no MMBGR, a partir das considerações da pesquisadora e dos autores do instrumento MBGR

**ITEM – MBGR –** **História Clínica (original)**	**MMBGR – História Clínica (itens modificados do MBGR)**
Dados pessoais	“frequenta creche/escola” / “turma” ao invés de “trabalha/já trabalhou”/ “ano”
Como e onde faz as refeições	“Brincando” ao invés de “Fazendo lição”
Capacidade Mastigatória	Grau de satisfação “da família”, ao invés de grau de satisfação do próprio “paciente”
Comunicação	“Elabora” ao invés de “Demorou a elaborar”

Na primeira análise dos especialistas, 70% do instrutivo, obteve concordância por pelo menos 70% destes; e 64% dos itens da “História Clínica” com concordância, por pelo menos 90% (Tabela 3). Para a segunda análise, foi submetida a versão final do instrumento já com todas as alterações sugeridas. Obteve-se, então, 100% de respostas “Concordo totalmente”. A seguir são descritos o Instrutivo (Apêndice 1) e o Protocolo de “História Clínica Miofuncional Orofacial” (Apêndice 2) que compõem, junto ao Exame Clínico, o “Protocolo MMBGR – Lactentes e Pré-Escolares”.

**Tabela 3:** Porcentagem de concordância entre os especialistas e Índice de validade de Conteúdo (IVC) referente aos dados específicos dos Instrutivo e História Clínica do Protocolo MMBGR

**Nº de Avaliadores de acordo**	**Nº de Itens (%)**	**IVC (%)**	**p-valor**
**Instrutivo**			
10	0 (0,0)	100	1,000
9	3 (30,0)	90	0,972
8	3 (30,0)	80	0,851
7	1 (10,0)	70	0,617
6	2 (20,0)	60	0,350
5	0 (0,0)	50	0,150
4	1 (10,0)	40	0,047
**História Clínica**			
10	26 (40,6)	100	1,000
9	15 (23,4)	90	0,972
8	13 (20,3)	80	0,851
7	8 (12,5)	70	0,617
6	1 (1,6)	60	0,350
5	1 (1,6)	50	0,150

Teste Binomial Exato

**Legenda:** IVC = Índice de Validade de Conteúdo; % = Frequência relativa percentual

O “**instrutivo”** contempla orientações sobre a utilização do tanto em relação à aplicação, como ao Registro nos Protocolos de “História Clínica Miofuncional Orofacial” (anamnese/ entrevista) e “Exame Clínico” (Exame miofuncional orofacial com escores). Também explicita sobre o objetivo principal do MMBGR, destinado ao fonoaudiólogo avaliar, diagnosticar e estabelecer prognóstico em MO; o tempo médio para a sua aplicação (30-45 minutos para “História Clínica”, e 60-90 minutos para o “Exame Clínico”), com demanda de cerca de 2 horas de trabalho para análises dos dados obtidos.

O instrutivo ainda fornece informações sobre o Exame Clínico: I. Procedimentos para coleta/análise do Exame Miofuncional Orofacial, incluindo padronizações de registro em fotografia/vídeo, roteiro de registro de imagens estáticas e dinâmicas; II. Procedimentos sobre itens a serem avaliados, faixas etárias e escores; III. Orientações gerais sobre aspectos do protocolo a serem considerados, de acordo com a faixa etária, incluindo material utilizado e forma de registro dos seguintes itens: Identificação e dados antropométricos, seguidos pelos itens do exame miofuncional orofacial: **exame extraoral** – análise facial subjetiva, lábios e mandíbula; **exame intraoral** – lábios, bochechas, língua, palato, tonsilas palatinas, dentes e oclusão; **tônus** – lábios, mento, língua e bochechas; **funções orofaciais** – Respiração, Sucção, Mastigação, Deglutição (líquido, pastoso e sólido/semissólido) e Fala.

O Protocolo para levantamento da **“História Clínica”** contempla aspectos a serem levantados junto ao responsável pelo lactente/pré-escolar na situação de anamnese/entrevista inicial. A maioria dos itens podem ser assinalados caso haja a ocorrência, e existem espaços para complementação de informações, descrições e observações. Contém itens relacionados desde a identificação do lactente/pré-escolar, tais como: número de registro, nome, datas (exame e nascimento), idade, informante, escolaridade, endereço, dados familiares e de contato), até dados relacionados diretamente à queixa e ao motivo do encaminhamento para a Fonoaudiologia.

Os aspectos sobre desenvolvimento estão organizados conforme ocorrência e período, além de registro das dificuldades encontradas; incluindo cronologia referencial sobre a aquisição de cada comportamento. Constam itens relacionados aos aspectos do Desenvolvimento e dificuldade motora, da Alimentação (desde amamentação até uso de utensílios), além de serem abordados aspectos voltados aos problemas de saúde, respiratórios, sono; bem como tratamentos fonoaudiológicos e em áreas interdisciplinares; e ocorrência de hábitos deletérios. Dados sobre o padrão atual do lactente/pré-escolar também estão elencados, incluindo aspectos alimentares, da mastigação, da deglutição, da fala, da comunicação oral e da voz.

## DISCUSSÃO

O Estudo foi conduzido com o propósito de adaptar e validar o conteúdo e a aparência do Protocolo de Avaliação Miofuncional Orofacial MBGR para aplicação em lactentes e pré-escolares. Para isso, a partir da experiência da prática profissional dos autores e do referencial bibliográfico consultado, foi construído o Instrutivo e elaboradas as versões dos “Protocolos de História Clínica” e “Exame Clínico Miofuncional Orofacial” com escores para serem utilizadas com a faixa etária de 6 meses a 5 anos e 11 meses de vida.

Considerou-se a elaboração do Instrutivo, para que este funcione como guia instrucional que oriente sobre o uso do protocolo, padronização dos registros e documentações, por parte do fonoaudiólogo. O guia instrucional é parte integrante do Protocolo MMBGR – Lactentes e Pré-escolares, e a sua utilização deve compor o raciocínio clínico inerente à aplicação dos protocolos de História Clínica e Exame Clínico.

Neste artigo foram apresentados apenas o Instrutivo e o Protocolo História Clínica Miofuncional Orofacial. A análise do conteúdo do protocolo possibilitou a manutenção, acréscimo e exclusão de itens e, considerando a escassez de protocolos validados na área de MO para menores de 6 anos de vida, os principais aspectos abordados foram extraídos da literatura referência de desenvolvimento infantil.

No protocolo MMBGR História Clínica foram acrescidos os dados sobre os irmãos, sendo considerada a constituição familiar um aspecto importante a ser compreendido no processo terapêutico^(4)^. Por outro lado, foram excluídos itens não condizentes com a faixa etária. Também foram acrescidos aspectos sobre Desenvolvimento motor global, visto que a progressão do controle corporal, evolui para um conjunto de aquisições, equilíbrio nas diferentes posturas e posições, que levam a criança à independência funcional^(17)^, fundamental para a situação de alimentação. Os padrões motores abordados e quais habilidades motoras alteradas basearam-se no *Alberta Infant Motor Scale* (AIMS): Valores de referência para categorização do desempenho motor de crianças^(17,18)^.

Os aspectos sobre Desenvolvimento da alimentação, com padrões por faixa etária, foram fundamentados no Guia alimentar para crianças brasileiras menores de 2 anos, do Ministério da Saúde do Brasil^(19)^, e em protocolos internacionais^(1,3)^, visto que não existem instrumentos validados no Brasil para essa faixa etária. O protocolo ChOMPS - *Child Oral and Motor Proficiency Scale* ^(1)^ foi consultado por ser instrumento que investiga, a partir dos relatos dos pais, aspectos sobre comer, beber e habilidades relacionadas à alimentação, como por exemplo, capacidade de morder alimentos macios e beber líquidos finos sem tossir ou engasgar, bem como habilidades motoras que suportam a deglutição segura e o comer independente, como por exemplo, a capacidade de se sentar na vertical sem suporte^(1)^.

Considerando a importância de detecção precoce de dificuldades alimentares, foram estudados os aspectos abordados por Thoyre et al. (2018), conforme o protocolo *Pediatric Eating Assessment Tool* - PediEAT^(3)^, o qual avalia sintomas de problemas alimentares, buscando identificar precocemente os problemas de alimentação, a partir de relato parental. Sobre a dificuldade em introduzir utensílios, foram consideradas as pesquisas^(20,21)^ cujos relatos das mães referem-se, entre outros aspectos, sobre a capacidade de o bebê beber de um copo (com e sem tampa), tomar com um canudo (grande e pequeno) e se a criança tem autonomia ou é assistida pela mãe quando usa o utensílio para beber. Dados semelhantes sobre colheres (tipo e uso) foram considerados na presente adaptação. Quanto à Amamentação, de modo geral preconiza-se o aleitamento materno exclusivo até 6 meses de vida, com continuidade até os 24 meses, complementando-se a dieta, a qual privilegia a introdução de novas consistências, que favoreçam o desenvolvimento do sistema estomatognático.

Os aspectos referentes à Comunicação e Fala foram pautados nos estudos sobre o desenvolvimento da fala^(22,23)^, considerando somente os aspectos de produção articulatória relacionada à MO, em idades iniciais.

Consideramos que os valores de concordância obtidos são positivos, equiparando-se a outros estudos que também realizaram análises de conteúdo e aparência de instrumentos da área de MO, por meio do cálculo do IVC^(11)^. Salientamos que, após a segunda análise, 100% dos especialistas descreveram “Concordo Totalmente” para o conteúdo e a aparência do novo instrumento. O instrutivo e protocolo de História Clínica Miofuncional Orofacial, integrantes do Protocolo MMBGR – Lactente e pré-escolares, preenche uma importante lacuna para a clínica da MO e suas pesquisas, ampliando o conhecimento científico na Fonoaudiologia. Novos estudos devem ser propostos para as demais etapas do processo de validação. O nosso grupo de pesquisa tem se debruçado na subsequente etapa da “evidência de validade baseada nos processos de resposta”, tal como recomendado na literatura^(7)^, para viabilizar a aplicabilidade na população em questão.

## CONCLUSÃO

A adaptação do Protocolo de Avaliação Miofuncional Orofacial MBGR para aplicação em lactentes e pré-escolares foi realizada e, a partir disso, o Instrutivo e o Protocolo História Clínica Miofuncional Orofacial teve a validação de conteúdo e aparência concluída, possibilitando a sua utilização para faixa etária de 6 meses a 5 anos e 11 meses de vida.

## Apêndice 1 Protocolo MMBGR – Lactentes e Pré-escolares - Instrutivo

PROTOCOLO MMBGR - Lactentes e Pré-Escolares

Andréa Monteiro Correia Medeiros, Irene Queiroz Marchesan, Katia Flores Genaro, Giédre Berretin-Felix

Instrutivo sobre a aplicação e registro

O Protocolo MMBGR – Lactentes e Pré-escolares é um instrumento da área de Motricidade Orofacial com escores, indicado para ser utilizado na população de 6 meses a 5 anos e 11 meses de vida.

Compõe-se dos protocolos de HISTÓRIA CLÍNICA (anamnese/entrevista) e de EXAME MIOFUNCIONAL OROFACIAL COM ESCORES (avaliação), destinado ao fonoaudiólogo avaliar, diagnosticar e estabelecer prognóstico em Motricidade Orofacial.

Para aplicá-lo é despendido um tempo médio de 30-45 minutos para HISTÓRIA CLÍNICA, e 60-90 minutos para coleta de dados do EXAME CLÌNICO, sendo que a análise dos resultados demanda cerca de 2 horas de trabalho.

Alguns domínios podem ser analisados por intermédio da História Clínica e outros pelo Exame Clínico, enquanto o raciocínio clínico é realizado pela compreensão da relação entre os dados registrados nos dois protocolos.

PROTOCOLO HISTÓRIA CLÍNICA:

Aplica-se com o responsável (principal cuidador) pelo lactente/pré-escolar, antes da realização do exame miofuncional orofacial.

Escolhe-se um ambiente privativo e tranquilo, sendo os dados colhidos diretamente pelo fonoaudiólogo e registrados no momento do levantamento, junto ao responsável.

Contém itens que compreendem: identificação, queixas, antecedentes familiares e intercorrências; desenvolvimento e dificuldades motoras; problemas de saúde geral e/ou específicos, tais como respiratórios, sono e tratamentos realizados; aspectos ligados à alimentação desde a amamentação até a alimentação atual, incluindo principais dificuldades e padrão de alimentação; bem como sobre a respiração, mastigação, deglutição, hábitos orais e, também aspectos sobre a fala, comunicação oral, audição e voz.

PROTOCOLO EXAME MIOFUNCIONAL OROFACIAL COM ESCORES:

Aplica-se diretamente junto ao lactente/pré-escolar, preferencialmente com a presença do responsável na sala durante o exame, principalmente em sujeitos na faixa etária até 23 meses de idade. Considera-se, ainda, que, a depender da idade e nível de compreensão da criança, as questões devem ser dirigidas a ela, em linguagem adequada para o seu entendimento e obtenção de resposta, com a concordância do responsável.

Escolhe-se um ambiente privativo e tranquilo, com boa iluminação, sendo que todos os dados podem ser coletados diretamente pelo fonoaudiólogo junto ao sujeito e devidamente registrados no protocolo no momento do levantamento (tempo real).

O registro do exame por meio de documentação fotográfica e em vídeo, para posterior certificação e análise dos dados previamente levantados, também é recomendado.

I. Procedimentos para posicionamento no Exame Miofuncional Orofacial e padronização de documentação:

Tanto na observação em tempo real, quanto durante a análise de registro em fotografia e/ou vídeo, o sujeito deve ser observado posicionado sentado de frente para o examinador, com as costas apoiadas, com correção da cabeça:

▪ Lactente (6 a 23 meses): no colo do responsável;

▪ Pré-escolar (24 a 71 meses): em uma cadeira, mantendo os pés em contato com o chão (cadeira adequada ao tamanho do sujeito). Até os 47 meses pode estar posicionado no colo do responsável.

Pode ser utilizada uma cadeira de tamanho padrão, e nesses casos, quando a altura do paciente não permitir o apoio plantar, usar um apoio para os pés que garanta um ângulo equivalente à cadeira menor.

O examinador deve estar sentado, de frente ao sujeito, mantendo seus olhos na altura dos olhos do lactente/pré-escolar. As indicações de posicionamento devem contemplar uma forma ergonômica adequada para a realização do exame para ambos (examinador e sujeito).

Para registro em fotografia/vídeo, devem ser observadas algumas padronizações:

▪ A distância entre o tripé da máquina fotográfica e o sujeito deve ser a mesma em todas as avaliações. Pode-se, inclusive, haver sinalizações no chão e paredes quanto aos planos e ângulos a serem padronizados para o posicionamento do sujeito, ao realizar os registros das imagens.

As distâncias para cada registro fotográfico e em vídeo podem variar de acordo com as necessidades do avaliador, bem como com as especificidades de cada equipamento utilizado (câmera fotográfica, flash e lentes), do espaço físico e da luminosidade da sala. (Frazão e Manzi, 2019)

Para documentar a região intraoral, as distâncias devem ser mais aproximadas entre o tripé e o sujeito, do que para o registro da face. Indica-se usar lentes Macro na câmera fotográfica (Frazão e Manzi, 2019).

O Protocolo apresenta o seguinte Roteiro recomendado para registro de imagens. Entretanto, a depender das necessidades do avaliador, como também da idade, capacidade de compreensão e execução do movimento por parte do sujeito, outras imagens podem ser registradas (imagens extras).

Referência: Frazão YS, Manzi SHB. Atualização em documentação fotográfica e em vídeo na motricidade orofacial. In: Silva HJ da, Tessitore A, Motta AR, Cunha DA da, Berretin-Felix G, Marchesan IQ, editors. Tratado de Motricidade Orofacial. 1st ed. São José dos Campos: Pulso Editorial; 2019. p. 243–53.

**Table:**

Roteiro para registo de imagens					
	Imagens Estáticas				
	- Face:	[ ] Visão frontal sem correção da postura da cabeça		[ ] Visão frontal com correção da postura da cabeça	
	- Lábios:	[ ] Em repouso – habitual	[ ] Mucosa interna	[ ] Frênulo labial superior	
	- Bochechas:	[ ] Mucosa interna direita	[ ] Mucosa interna esquerda		
	- Língua:	[ ] Exteriorizada *(fora da cavidade oral)*			
		[ ] Frênulo *(língua elevada sem tocar o palato)*		[ ] Frênulo *(língua elevada com manobra)*	
	- Palato:	[ ] Duro			
	- Dentes:	[ ] Arcada Superior	[ ]Arcada Inferior		
	- Oclusão:	[ ] Anterior	[ ] Lado Direito	[ ] Lado Esquerdo	
	- Outras:	[ ] À critério do examinador			
			
	**Imagens Dinâmicas**				
	- Sucção:	[ ] Amamentação *(peito)*	[ ] Mamadeira		
	- Mastigação:	[ ] Boca aberta após mastigar e antes de deglutir			
	- Deglutição:	[ ] Líquido	[ ] Pastoso	[ ] Sólido/Semissólido	[ ] Boca aberta após deglutir *(resíduo)*
	- Fala:	[ ] Semi-dirigida	[ ] Nomeação de figuras/repetição		
	- Orofaringe:	[ ] Palato mole	[ ] Úvula	[ ] Tonsilas Palatinas	

II. Procedimentos sobre itens a serem avaliados, faixas etárias e escores:

O Protocolo MMBGR Lactentes/Pré-escolares abrange itens de identificação e dados antropométricos, seguidos pelos itens do exame miofuncional orofacial: **exame extraoral** – análise facial subjetiva, lábios e mandíbula; **exame intraoral** – lábios, bochechas, língua, palato, tonsilas palatinas, dentes e oclusão; **tônus** – lábios, mento, língua e bochechas; **funções orofaciais** – Respiração, Sucção, Mastigação, Deglutição (líquido, pastoso e sólido/semissólido) e Fala.

A maioria dos itens são obtidos para todas as faixas etárias, enquanto outros seguem a indicação de faixa etária informada no próprio instrumento. Isso porque, todo o instrumento foi elaborado respeitando-se o desenvolvimento esperado, e possível de ser realizado, em cada faixa etária abordada. Entretanto, cada item pode ser aplicado além ou aquém das idades sugeridas, de acordo com o desenvolvimento individual da criança.

Para fins de análise são atribuídos escores por faixa etária em meses, para cada item avaliado, sendo que ao final, os escores devem ser registrados no quadro de Resumo de Exame Miofuncional Orofacial. Escores mais altos estão relacionados aos piores padrões de motricidade orofacial observado no lactente e pré-escolar. No quadro resumo estão descritos os melhores e piores resultados possíveis de serem obtidos por grupo etário. Entretanto, esses valores não devem ser adotados enquanto nota de corte para diagnóstico, mas podem servir como valores de referência individual, durante o acompanhamento/ processo de reabilitação de cada paciente.

III. Orientações gerais sobre aspectos do protocolo a serem considerados, de acordo com a faixa etária:

**Table:**

**1. Identificação**			
✓Todas as faixas etárias. ✓Dados obtidos diretamente com o responsável, e/ou extraídos da caderneta de saúde da criança.			
**2. Exame Extraoral**			
✓Todas as faixas etárias. ✓Avaliação in loco e/ou registro em imagem *(foto)* em visão frontal, para análise posterior ao exame miofuncional orofacial.			
**3. Exame Intraoral**			
✓Todas as faixas etárias *(escores diferenciados para lactentes e pré-escolares, a depender do item avaliado)* ✓Avaliação in loco e/ou registro em imagem *(foto)* com lente macro, para análise posterior ao exame miofuncional orofacial. ✓Examinador deve realizar a inspeção intraoral do sujeito, usando luvas de procedimento, tal como no exame do frênulo da língua; usando afastador labial para avaliação de dentes e oclusão *(quando houver dificuldade de colocação do afastador labial em crianças de seis meses a quatro anos de idade, solicita-se que os lábios sejam afastados com os dedos do responsável).* ✓O examinador também deve solicitar para que a criança abra a boca de forma voluntária e/ou por imitação, para observação de estruturas como língua, palato e tonsilas palatinas.			
**4. Tônus**			
✓Todas as faixas etárias. ✓Examinador deve realizar obrigatoriamente observação visual e palpação das estruturas. ✓A impressão do resultado, a partir do exame de palpação deve ser registrada em tempo real, e posteriormente confirmada, quando possível, a partir da imagem realizada.			
**5. Funções orofaciais**			
✓Para cada função orofacial relacionada à alimentação, utilizar sempre o mesmo tipo de alimento, observando sua consistência. Pode-se abrir exceções quanto ao tipo de alimento, de acordo com o hábito e aceitação/recusa individual do sujeito. ✓Preconiza-se sempre utilizar o alimento trazido pela família. ✓Na avaliação da Fala, para nomeação de figuras, indica-se utilizar o material pertencente ao instrumento *(Protocolo MMBGR – Figuras para Nomeação).* ✓Registro em vídeo *(filmagem)* é recomendado para ser utilizado na análise posterior ao exame miofuncional orofacial.			
Orientações gerais sobre os aspectos a serem documentados/avaliados: Funções Orofaciais			
**Função Orofacial**	**Faixa etária**	**Material utilizado**	**Exame/ Registro específico**
Respiração	▪Todas (6 a 71 meses)	▪Espelho de Glatzel/ ▪Espelho nasal milimetrado de Altmann	▪Mensurar fluxo aéreo, em tempo real ▪Não é obrigatória a limpeza com soro fisiológico, mas recomenda-se que o sujeito assoe o nariz antes do procedimento. ▪Marcar fluxo aéreo em papel milimetrado
Sucção/Deglutição	▪Até 23 meses, desde que ainda seja amamentado	▪Peito *(leite materno)*	▪Observar mamada no peito *(2 a 5 minutos)*
Sucção/Deglutição	▪Até 23 meses, desde que ainda utilize mamadeira na sua rotina	▪Mamadeira *(leite materno ou fórmula láctea)*	▪Observar oferta de mamadeira *(3 minutos ou pelo menos 10 ml)*
Mastigação	▪A partir de 12 meses	▪Mão/ talheres *(pão francês ou biscoito)*	▪Observar por 2 minutos, ou dois ciclos completos, até ocorrer a deglutição. ▪A partir de 36 meses solicitar que o sujeito abra a boca antes de deglutir, para verificar a eficiência da trituração do alimento.
Deglutição - líquido	▪A partir de 12 meses	▪Copo *(água, suco, ou leite materno/fórmula)*	▪Observar o esvaziamento de pelo menos ¼ de copo *(50 ml).*
Deglutição - pastoso	▪Até 11 meses ▪Até 23 meses, quando se alimentam na consistência pastosa	▪Colher *(papa, purê, alimento amassado, tal como banana)*	▪Observar por 2 minutos, ou um ciclo completo, até concluir a deglutição *(elevação laríngea)*
Deglutição - sólido/semissólido	▪A partir de 12 meses	▪Mão/talheres *(pão francês, biscoito, entre outros)*	▪Observar por 2 minutos, ou um ciclo completo, até concluir a deglutição *(elevação laríngea)* ▪Solicitar que o sujeito abra a boca após deglutir, para verificar resíduo de alimento.
Fala - Produção de fones e fonemas	▪A partir de 24 meses	▪Lista de figuras *(Protocolo MMBGR - Figuras para Nomeação)* ▪Fala semidirigida	▪Nomeação de Figuras: o sujeito tem que dizer o nome da figura mostrada. ▪Caso não saiba, o examinador nomeia a figura, e depois de mostrar outra figura da lista, volta a mostrar a figura não nomeada inicialmente. Pede-se então para o sujeito nomeá-la. ▪Após isso, não será dada nova chance. Apenas registra-se que o sujeito não conseguiu produzir mesmo com a tentativa de repetição do examinador. ▪Fala semi-dirigida: pode ser observada durante todo o exame miofuncional orofacial e, também pedindo para o sujeito dizer o nome e idade; falar sobre a escola ou uma brincadeira; contar sobre uma viagem ou passeio ▪Elaboração do quadro fonêmico, a partir da produção do sujeito.
Fala - Aspectos gerais da Fonoarticulação	▪A partir de 36 meses	▪Fala semidirigida	▪Fala semidirigida: pode ser observada durante todo o exame miofuncional orofacial e, também pedindo para o sujeito dizer o nome e idade; falar sobre a escola ou uma brincadeira; contar sobre uma viagem ou passeio

## Apêndice 2 Protocolo MMBGR - Lactentes e pré-escolares: história clínica miofuncional orofacial

PROTOCOLO MMBGR - LACTENTES E PRÉ-ESCOLARES (6 meses até 5 anos e 11 meses)

Andréa Monteiro Correia Medeiros, Irene Queiroz Marchesan, Katia Flores Genaro, Giédre Berretin-Felix

**Table:**

**Nome**: ___________________________________________		**Nº**: ________________
**Data do exame**: ___ / ___ / ___	**Idade:** _____ anos e _____ meses	**DN:** ____ / ____ / ____
**Informante:** _______________________	**Grau de parentesco:** ________________________________________	

**Table:**

**Frequenta creche/escola:** ❒ não ❒ sim	**Turma:** _________	**Período:**❒ matutino ❒ vespertino ❒ integral

**Table:**

**Endereço:**________________________________________________	**Nº:**________	**Complemento:**____________
**Bairro:** ___________________________	**Cidade/Estado:**__________________	**CEP:**_____________________

**Table:**

**Filiação:**	Nome do Pai: __________________________________		Nome da Mãe: ____________________________
	E-mail: _______________________________________		E-mail: __________________________________
	Telefone Celular: ( ___ ) ___________________________		Telefone Celular: ( ___ ) ______________________
	Telefone do Trabalho: ( ___ ) _______________________		Telefone do Trabalho: ( ___ ) __________________
**Irmãos:**	❒ não	❒ sim - Quantidade: _____________________	Idade dos irmãos: _________________________

**Table:**

**Quem indicou para Fonoaudiologia?** *(nome, especialidade, telefone):* *____________________________________________________________________________________________________*
**Por qual motivo?** _____________________________________________________________________________________

**Table:**

**Queixa principal (do responsável):** ______________________________________________________________________

**Table:**

**Outras queixas relacionadas:**						
❒ lábios	❒ dentes	❒ frênulo lingual	❒ sucção	❒ mastigação	❒ fala	❒ postura corporal
❒ língua	❒ oclusão	❒ deglutição	❒ respiração	❒ voz	❒ audição	

Informações adicionais: ____________________________________________________________________________________________

**Table:**

**Antecedentes familiares (em relação à queixa):**	
❒ não	❒ sim - Qual: _________________________________________________________________________________

**Table:**

**Intercorrências:**		
Na gestação:	❒ não	❒ sim - Qual: _____________________________________________________________
No nascimento:	❒ não	❒ sim - Qual: _____________________________________________________________

**Table:**

**Desenvolvimento motor** (*assinalar se já realizou o comportamento e em qual idade*)**:**				Não sabe/ Não lembra		Idade Esperada
Sustentar a cabeça	❒ não	❒ sim: ___ meses		❒		3 meses
Primeiras tentativas de locomoção: arrastar, rastejar	❒ não	❒ sim: ___ meses		❒		6 a 7 meses
Sentar independente	❒ não	❒ sim: ___ meses		❒		7 meses
Brincar em decúbito lateral	❒ não	❒ sim: ___ meses		❒		7 meses
Rolar	❒ não	❒ sim: ___ meses		❒		7 meses
Sentar sem suporte (utiliza mãos: atividade exploratória)	❒ não	❒ sim: ___ meses		❒		8 meses
Sentar estavelmente (utiliza mãos: manipula e explora objeto)	❒ não	❒ sim: ___ meses		❒		9 meses
Engatinhar	❒ não	❒ sim: ___ meses		❒		10 meses
Ficar em pé	❒ não	❒ sim: ___ meses		❒		10 meses
Andar sem apoio	❒ não	❒ sim: ___ meses		❒		13 meses
De modo geral, desenvolvimento motor pode ser considerado	❒ normal	❒ alterado				
Se alterado, assinale em quais habilidades:	[ ] sustentar a cabeça		[ ] sentar		[ ] ficar em pé	
	[ ] rolar		[ ] engatinhar		[ ] andar sem apoio	

**Table:**

**Dificuldade motora:**	❒ não	❒ sim:	[ ] correr	[ ] andar de triciclo/bicicleta com rodinhas de apoio	[ ] vestir-se
			[ ] amarrar os sapatos	[ ] abotoar	[ ] escovar os dentes
			[ ] pintar (segurar lápis)	[ ]Usar colher/garfo	[ ]outras: _________________

**Table:** Problemas de saúde

			Descrever	Tratamento/Medicamento
Neurológico:	❒ não	❒ sim:	_____________________	_______________________________________
Ortopédico:	❒ não	❒ sim:	_____________________	________________________________________
Metabólico:	❒ não	❒ sim:	_____________________	________________________________________
Digestivo:	❒ não	❒ sim:	_____________________	________________________________________
Hormonal:	❒ não	❒ sim:	_____________________	________________________________________
Otorrinolaringológico:	❒ não	❒ sim:	_____________________	________________________________________
Pneumológico:	❒ não	❒ sim:	_____________________	________________________________________
Cardiológico:	❒ não	❒ sim:	_____________________	________________________________________
Emocional/Psíquico:	❒ não	❒ sim:	_____________________	________________________________________

Outros problemas: ________________________________________________________________________________________________________________

**Table:** Problemas respiratórios

			Tratamento/Medicamento
Resfriados frequentes*****:	❒ não	❒ sim:	_________________________________________________________
Problemas de garganta:	❒ não	❒ sim:	________________________________________________________________
Amidalite:	❒ não	❒ sim:	________________________________________________________________
Halitose:	❒ não	❒ sim:	________________________________________________________________
Asma:	❒ não	❒ sim:	________________________________________________________________
Bronquite:	❒ não	❒ sim:	________________________________________________________________
Pneumonia:	❒ não	❒ sim:	________________________________________________________________
Rinite:	❒ não	❒ sim:	________________________________________________________________
Sinusite:	❒ não	❒ sim:	________________________________________________________________
Obstrução nasal:	❒ não	❒ sim:	________________________________________________________________
Prurido nasal:	❒ não	❒ sim:	________________________________________________________________
Coriza:	❒ não	❒ sim:	________________________________________________________________
Espirros em salva:	❒ não	❒ sim:	________________________________________________________________

* *resfriado frequente (alteração de via aérea superior) até 5 anos de idade: acima de 12 episódios/ano*

Outros problemas: _____________________________________________________________________________________________

**Table:** Sono

Horário que dorme: _____	Horário que acorda: _____		
Noturno: ❒< 8 horas	❒> 8 horas: descrever a frequência ____________________________________		
Dorme de dia:	❒ não	❒ sim:	_______________________________________________
Agitado:	❒ não	❒ sim:	_______________________________________________
Fragmentado:	❒ não	❒ sim:	_______________________________________________
Enurese noturna:	❒ não	❒ sim:	_______________________________________________
Ronco:	❒ não	❒ sim:	_______________________________________________
Ressona:	❒ não	❒ sim:	_______________________________________________
Sialorreia:	❒ não	❒ sim:	_______________________________________________
Apneia:	❒ não	❒ sim:	_______________________________________________
Bruxismo:	❒ não	❒ sim:	_______________________________________________
Ingestão de água à noite:	❒ não	❒ sim:	_______________________________________________
Boca aberta ao dormir:	❒ não	❒ sim:	_______________________________________________
Boca seca ao acordar:	❒ não	❒ sim:	_______________________________________________
Mão posicionada sob o rosto:	❒ não	❒ sim: [ ] D [ ] E _______________________________________________	
Postura: ❒ decúbito lateral	❒ decúbito dorsal		❒ decúbito ventral

Outros problemas: ___________________________________________________________________________________________________________________

**Table:** Tratamentos

				Nome/Contato
**Fonoaudiológico:**	❒ não	❒ no passado	❒ atualmente	
Motivo: ________________________________________				___________________________
**Médico:**	❒ não	❒ no passado	❒ atualmente	
Qual(is) especialidade(s): _________________________				
Motivo: ________________________________________				___________________________
**Psicológico:**	❒ não	❒ no passado	❒ atualmente	
Motivo: ________________________________________				__________________________
**Fisioterápico:**	❒ não	❒ no passado	❒ atualmente	
Motivo: ________________________________________				__________________________
**Odontológico:**	❒ não	❒ no passado	❒ atualmente	
Motivo: ________________________________________				__________________________
Tipo de Procedimento: ____________________________				
**Cirúrgico:**	❒ não	❒ no passado	Quando? _______	
Motivo: ________________________________________				__________________________
Tipo de Procedimento: ____________________________				

Outros tratamentos: _______________________________________________________________________________________________

**Table:** Desenvolvimento da alimentação Amamentação

Peito (Aleitamento materno):	❒ não	❒ sim	
Exclusivo:	❒ não	❒ sim - Até quando: _______________	❒ não sabe/não lembra
Complementado:	❒ não	❒ sim - Até quando: _______________	❒ não sabe/não lembra

**Table:** Alimentação – dificuldade em introduzir

Utensílios:	❒ canudo	❒ copo	❒ colher	❒ garfo	Descrever: _____________________
Sabores:	❒ salgado	❒ doce	❒ azedo	❒ amargo	Descrever: _____________________
Consistências:	❒ líquida	❒ pastosa	❒ semissólida	❒ sólida	Descrever: _____________________

**Table:** **Utensílio utilizado atualmente para oferta da alimentação** (pode assinalar mais de uma alternativa)**:**

❒ mamadeira – tipo de bico: _______________ material: _______________ furo: _______________			
❒ copo comum (aberto)	❒ copo com tampa	❒ copo com válvula	❒ copo com canudo
❒ colher	❒ garfo		❒ outro ____________________

**Table:** Padrão alimentar atual

**Comportamento de prontidão e padrão de alimentação**			**Não sabe/** **Não lembra**	**Idade** **esperada**
Abre a boca em direção à colher quando está com fome	❒ não	❒ sim	❒	6 meses
Inclina-se para frente em direção à colher, segurando a mão da pessoa que oferece o alimento	❒ não	❒ sim	❒	6 meses
Aceita alimentos raspados e/ou amassados com colher	❒ não	❒ sim	❒	6 a 7 meses
Inclina-se para a colher, pega ou aponta o alimento	❒ não	❒ sim	❒	7 a 11 meses
Aceita alimentos amassados com garfo, contendo pedaços pequenos	❒ não	❒ sim	❒	7 a 8 meses
Aceita alimentos picados ou em pedaços pequenos	❒ não	❒ sim	❒	9 a 11 meses
Come de forma independente, mas ainda precisa de ajuda	❒ não	❒ sim	❒	9 a 12 meses
Utiliza/combina palavras e gestos para expressar o desejo pelo alimento	❒ não	❒ sim	❒	12 a 24 meses
Aceita alimentos na consistência dos adultos	❒ não	❒ sim	❒	a partir de 12 meses
Come de forma independente com colher	❒ não	❒ sim	❒	12 a 24 meses
Segura o copo com ambas as mãos	❒ não	❒ sim	❒	12 a 24 meses
Demonstra habilidade para mastigar	❒ não	❒ sim	❒	12 a 24 meses

**Table:** Alimentação atual

**Tipo**		Descrever a frequência	Quais?
Frutas:	❒não	❒ sim: _______________	___________________________________
Verduras:	❒não	❒ sim: _______________	___________________________________
Legumes:	❒não	❒ sim: _______________	___________________________________
Cereais: *(arroz, macarrão, trigo)*	❒não	❒ sim: _______________	___________________________________
Grãos: *(feijão, lentilha, ervilha)*	❒não	❒ sim: _______________	___________________________________
Carnes:	❒não	❒ sim: _______________	___________________________________
Leite e derivados:	❒não	❒ sim: _______________	___________________________________
Açúcares:	❒ não	❒ sim: _______________	___________________________________

**Table:** De maneira geral, ingere alimentos predominantemente:

Preferência do sujeito:	❒ líquidos	❒ pastosos	❒ sólidos	❒ diferentes consistências
Ofertada pelo cuidador/ responsável:	❒ líquidos	❒ pastosos	❒ sólidos	❒ diferentes consistências

**Table:** Na maioria das vezes, com quem, como e onde faz as refeições:

Com qual companhia:	❒ sozinho	❒ família	❒ colegas *(escola)*		❒ outro - qual: _________________
Sem outra atividade:	❒ à mesa	❒ no sofá	❒ no chão	❒ na cama	❒ outro - qual: _________________
Assistindo televisão	❒ à mesa	❒ no sofá	❒ no chão	❒ na cama	❒ outro - qual: _________________
Brincando:	❒ à mesa	❒ no sofá	❒ no chão	❒ na cama	❒ outro - qual: _________________
Utilizando tablet/celular:	❒ à mesa	❒ no sofá	❒ no chão	❒ na cama	❒ outro - qual: _________________
Realizando outras atividades:	❒ à mesa	❒ no sofá	❒ no chão	❒ na cama	❒ outro - qual: _________________

**Table:** Mastigação

Lábios:	❒ fechados	❒ entreabertos	❒ abertos		
Ruído:	❒ não	❒ às vezes	❒ sim		
Dor ou desconforto durante a mastigação:			❒ não	❒ às vezes	❒ sim - [ ] direita [ ] esquerda
Possível causa da dor ao mastigar: ___________________________________________________________________________________					
Dificuldade mastigatória:		❒ não	❒ sim - Qual: ________________________________________________________		
Escape de alimentos durante a mastigação:			❒ não	❒ sim	
Necessita ingerir líquido junto aos alimentos:			❒ não	❒ sim	

Outros problemas: _____________________________________________________________________________________________________

**Table:** Mastiga os alimentos:

	adequadamente	pouco	muito	Não sabe informar sabe/não observou
Em comparação à família:	❒	❒	❒	❒
Em comparação aos amigos:	❒	❒	❒	❒
Descrever: ____________________________________________________________________________________________				

**Table:** Velocidade mastigatória

	semelhante	mais rápido	mais devagar	Não sabe informar observou
Em comparação à família:	❒	❒	❒	❒
Em comparação aos amigos:	❒	❒	❒	❒
Descrever: ____________________________________________________________________________				

**Table:** Capacidade mastigatória *(grau de satisfação da família do paciente com relação à sua mastigação)*

❒ ótima	❒ boa	❒ regular	❒ ruim

**Table:** Deglutição

			Descrever a frequência
Ruído:	❒ não	❒ sim	___________________________________________________________________________
Engasgos:	❒ não	❒ sim	________________________________________________________
Dor ao engolir:	❒ não	❒ sim	________________________________________________________
Refluxo nasal:	❒ não	❒ sim	________________________________________________________
Escape anterior:	❒ não	❒ sim	________________________________________________________
Pigarro:	❒ não	❒ sim	_________________________________________________________
Tosse:	❒ não	❒ sim	_________________________________________________________
Resíduos após a deglutição:	❒ não	❒ sim	________________________________________________________
Outras dificuldades:	❒ não	❒ sim	_________________________________________________________

Outros problemas: ____________________________________________________________________________________________________

**Table:** Hábitos orais

Sucção de chupeta:	❒ não	❒ sim - até quando: _______	[ ] comum	[ ] ortodôntica	[ ] não sabe informar
Sucção de dedo:	❒ não	❒ sim - até quando: _______			
Sucção de língua:	❒ não	❒ sim - até quando: _______			
Umidificar os lábios:	❒ não	❒ sim - idade: ___________			
Outros:_________________________________________			até quando: ____________________________		

**Table:** Hábitos de mordida

Bruxismo (ranger os dentes):	❒ não	❒ sim - [ ] diurno	
Apertamento dentário:	❒ não	❒ sim - até quando: _______	
Onicofagia (roer as unhas):	❒ não	❒ sim - até quando: _______	
Morder/ mordiscar lábios:	❒ não	❒ sim - até quando: _______	
Morder/Mordiscar mucosa interna das bochechas:		❒ não	❒ sim - até quando: _____________________
Morder objetos: Qual? _____________________		❒ não	❒ sim - até quando: _____________________

**Table:** Hábitos de postura

Interposição do lábio inferior:	❒ não	❒ sim
Interposição de língua:	❒ não	❒ sim
Protração da mandíbula:	❒ não	❒ sim
Apoio da mandíbula na mão:	❒ não	❒ sim [ ] direita [ ] esquerda
Apoio da cabeça na mão:	❒ não	❒ sim [ ] direita [ ] esquerda

Outros hábitos: _______________________________________________________________________________________________________

**Table:** Comunicação oral

Intencionalidade prejudicada:	❒ não	❒ sim - detalhar: _____________________________________	
Ausência de produção de sons:	❒ não	❒ sim - detalhar: _____________________________________	
Dificuldade de compreensão:	❒ não	❒ sim - detalhar: _____________________________________	
Demorou para começar a falar:	❒ não	❒ sim - detalhar: _____________________________________	
Dificuldade na elaboração de frases:		❒ não	❒ sim - detalhar: _____________________________
Gagueira *(hesitação/repetição/prolongamento)*:		❒ não	❒ sim - detalhar: _____________________________

OBS: No caso de suspeita de alteração, aplicar protocolo específico de linguagem

Outros problemas: ____________________________________________________________________________________________________________________________________________________________________________________________________________________________________________________________________________________________________________________________________________________________

**Table:** Fala

Omissão:	❒ não	❒ sim - descrever a frequência: _____________________________________________________		
Substituição:	❒ não	❒ sim - descrever a frequência: _____________________________________________________________		
Distorção:	❒ não	❒ sim - descrever a frequência: _____________________________________________________________		
Salivação excessiva:		❒ não	❒ sim - descrever a frequência: _________________________________________	
Interposição da língua:		❒ não	❒ sim - [ ] anterior [ ] lateral	
Abertura da boca:		❒ normal	❒restrita	❒ exagerada
Inteligibilidade prejudicada: ❒ não			❒ sim - descrever a frequência: _________________________________________	
Quais fones: ______________________________________________________________________________________________________				

Outros problemas: __________________________________________________________________________________________________________________________________________________________________________________________________________________________________________

**Table:** Audição

Hipoacusia *(diminuição da audição)*:	❒ não	❒ às vezes	❒ sim [ ] direita [ ] esquerda
Otite *(infecção de ouvido)*:	❒ não	❒ às vezes	❒ sim [ ] direita [ ] esquerda
Otalgia *(dor de ouvido)*:	❒ não	❒ às vezes	❒ sim [ ] direita [ ] esquerda
Avaliação audiológica prévia:	❒ não	❒ sim - Quando:_________________________________________________	
Resultados: _______________________________________________________________________________________________________			

Outros problemas: __________________________________________________________________________________________________________________________________________________________________________________________________________________________________________

**Table:** Voz

Rouquidão:	❒ não	❒ às vezes	❒ sim
Fraqueza:	❒ não	❒ às vezes	❒ sim
Afonia:	❒ não	❒ às vezes	❒ sim
Hipernasalidade:	❒ não	❒ às vezes	❒ sim
Hiponasalidade:	❒ não	❒ às vezes	❒ sim
Abuso vocal:	❒ não	❒ às vezes	❒ sim
Detalhar: _____________________________________________________________________________			

OBS: No caso de suspeita de alteração, aplicar protocolo específico vocal

Outros problemas: __________________________________________________________________________________________________________________________________________________________________________________________________________________________________________

Informações complementares:

______________________________________________________________________________________________________________________________________________________________________________________________________________________________________________________________________________________________________________________________________________________________________________________________________________________________________________________________________________________________________________________________________________________________________________________________________________________________________________________________________________________________________________________________

**Table:**

Fonoaudiólogo: _____________________________________________	CRFª:___________________________
