# Supplementary material for: ERRATUM: MMBGR Protocol - infants and preschoolers: instructive and orofacial myofunctional clinical history
Source: Codas. 2023 Jan 6;35(1):e20220159. doi: 10.1590/2317-1782/20212022159en (PMC10010419; doi:10.1590/2317-1782/20212022159en)
Supplement: Translation

# Translation: ERRATA: Protocolo MMBGR – lactentes e pré-escolares: instrutivo e história clínica miofuncional orofacial

**DOI:** 10.1590/2317-1782/20212022159pt

Devido ao erro honesto dos autores, o artigo “Protocolo MMBRG – lactentes e pré-escolares: instrutivo e história clínica miofuncional orofacial” (DOI https://doi.org/10.1590/2317-1782/20212020324), publicado em CoDAS 2022;34(2):e20200324, foi publicado com erros.

**No título em português, onde se lê:**

Protocolo MMBRG – lactentes e pré-escolares: instrutivo e história clínica miofuncional orofacial

**Leia-se:**

Protocolo MMBGR – lactentes e pré-escolares: instrutivo e história clínica miofuncional orofacial

**Na versão em inglês, nas páginas 4, 8, 9 e 12, onde se lê:**

Pasty

**Leia-se:**

Pudding

Os autores pedem desculpas pelos erros.
